# Metatranscriptomics Unravel Composition, Drivers, and Functions of the Active Microorganisms in Light-Flavor Liquor Fermentation

**DOI:** 10.1128/spectrum.02151-21

**Published:** 2022-05-31

**Authors:** Yuanyuan Pan, Ying Wang, Wenjun Hao, Chengbao Duan, Shiyuan Wang, Jinwang Wei, Gang Liu

**Affiliations:** a State Key Laboratory of Mycology, Institute of Microbiology, Chinese Academy of Sciences, Beijing, China; b Beijing Shunxin Agriculture Company Limited, Niulanshan Distillery, Beijing, China; c University of Chinese Academy of Sciences, Beijing, China; Tufts University

**Keywords:** metatranscriptomic analysis, active microbial community, drivers, active microbial function, light-flavor liquor fermentation

## Abstract

The microbial community in the fermented pit determines the quantity and quality of light-flavor liquor. Genetic diversity and the potential functions of the microbial community are often analyzed by DNA-based omics sequencing. However, the features of the active microbial community have not been systematically studied. Here, metatranscriptomic analysis was performed to elucidate the active microbial composition, drivers, and their functions in light-flavor liquor fermentation. Bacterial genera, *Lactobacillus*, Streptococcus, *Pediococcus*, *Thermotoga*, and *Faecalibacterium*, and fungal genera, *Saccharomyces*, *Talaromyces*, Aspergillus, *Clavispora*, *Rhizophagus*, *Cyberlindnera*, and *Wickerhamomyces*, were the dominant active microorganisms during the fermentation process. Additionally, they dominated the three-stage fermentation successively. Redundancy analysis showed that pH, ethanol, moisture, and starch were the main driving forces of microbial succession. Among the genes for the respective carbohydrate-active enzyme families, those for the glycoside hydrolase family 23, the glycosyltransferase family 2, the carbohydrate-binding module family 50, the polysaccharide lyase family 4, the auxiliary activity family 1, and the carbohydrate esterase family 9 showed the highest expression level. Additionally, the highly expressed enzymes and their contributed microorganisms were found in the key KEGG pathways, including carbohydrate metabolism, energy metabolism, lipid metabolism, and amino acid metabolism. Based on these data, a functional model of carbohydrate hydrolysis, ethanol production, and flavor generation were proposed. Taken together, *Saccharomyces*, *Lactobacillus*, *Wickerhamomyces*, *Pediococcus*, *Candida*, and *Faecalibacterium* were suggested as the core active microorganisms. Overall, our findings provide new insights into the composition, drivers, and functions of the active microorganisms, which is crucial for improving the quality of light-flavor liquor.

**IMPORTANCE** There is an urgent need for discovering the diversity and functions of the active microbial community in solid-state fermentation, especially in the pit of Chinese distilled liquor fermentation. Although the genetic composition of the microbial community has been clarified frequently by DNA-based sequencing, the composition and functions of the active microbial community have not been systematically revealed so far. Therefore, analysis of RNA-based data is crucial for discovering the functional microbial community. In this study, we employed metatranscriptomic analysis to elucidate the active microbial composition, successive drivers, and their functions in light-flavor liquor fermentation. The strategy can be broadly useful for discovering the active microbial community and exploring their functions in other types of flavor distilled liquor or other ecosystems. This study provides new insights into the understanding of the active microbial community composition and its functions.

## INTRODUCTION

Solid-state fermentation is considered one of the classical food fermentation strategies (1). Various microorganisms work together and interact with each other to produce abundant metabolically active compounds and special flavors (2). The traditional Chinese distilled liquor is a typical representative of that fermented food (3). The microbial community involved in distilled liquor fermentation is characterized by high species diversity, low culturability, and inability to simulate (4). Therefore, the intricate relationship between microorganisms and the fermentation process is still poorly understood.

The knowledge of microbial composition and its function is increasing concomitantly with the development of different molecular approaches, such as amplicon and high-throughput sequencing (5). Recently, metagenomic analysis of the microbial composition in the fermentation habitats has been employed to reveal the predominant microorganisms, analyze the sequential manner of the microbial community, and construct the correlation between the flavor compounds and the microbial members (6–9). Although metagenomic analysis provides extensive information about microbial composition, gene pools, and their potential functions, it is unable to distinguish whether these genes are expressed (10). Metatranscriptomic analysis, which is used for direct analysis of mRNA, provides a powerful tool to determine the active microbial members and their functions in natural habitats. Because of its advantages, metatranscriptomic analysis has been applied to reveal microbial composition and diversity in many fields, such as water and soil studies (11–13). Metatranscriptomic analysis has also been applied to study the gene expression of microorganisms in the distilled strong-flavor liquor (SFL) and Jiang-flavor liquor (JFL) fermentation (14–17). However, the active microbial composition, succession, drivers, and functions have not been clarified systematically so far in light-flavor liquor (LFL) fermentation.

In this study, metatranscriptomic analysis was used to comprehensively explore the composition, succession, drivers, and functions of the active microorganisms in LFL fermentation. The active microbial community displayed an obvious succession during the fermentation process. The drivers of microbial succession were determined by redundancy analysis (RDA). Additionally, the key KEGG pathways, including carbohydrate metabolism, energy metabolism, lipid metabolism, and amino acid metabolism were analyzed to find the highly expressed enzymes and the contributed microorganisms. Lastly, a model of carbohydrate hydrolysis, ethanol production, and flavor formation in LFL fermentation was proposed. Based on our study, the genera *Saccharomyces*, *Lactobacillus*, *Wickerhamomyces*, *Pediococcus*, *Candida*, and *Faecalibacterium* were suggested as the core active microorganisms in LFL fermentation. This study is valuable for initiating exquisite utilization of the core active microorganisms and improving the quality of LFL.

## RESULTS

### Metatranscriptomic sequencing and *de novo* assembly.

We obtained 387.99 Gbp of the raw data from 2,751,785,770 reads in total by metatranscriptomic sequencing, and then 377.27 Gbp of the clean data from 2,706,179,902 reads were used for the subsequent analysis. The Q20 value of all samples exceeded 98.21%, indicating the sequences had high accuracy (Table S1). The assembly quality was shown in Table S2. The contig numbers of all samples ranged from 5,291 to 14,639. The longest contig lengths ranged from 5,686 bp to 54,864 bp, and the N_50_ lengths ranged from 870 bp to 1,771 bp. The numbers and average length of unigenes were 36,834 and 1,304 bp, respectively (Table S3).

### Composition of the predominant active microorganisms.

**FIG 1:**
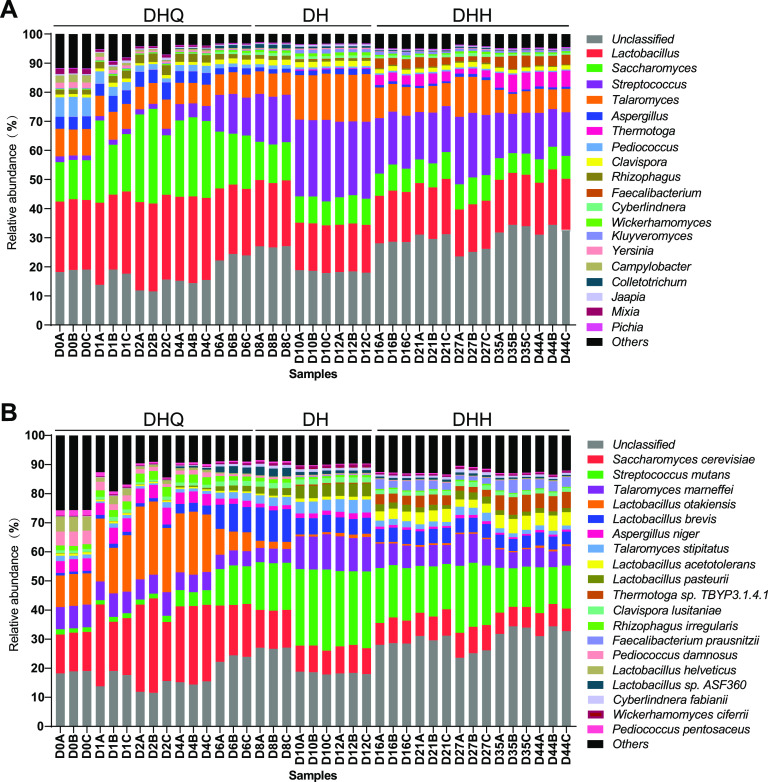
Composition of the active microbial community in light-flavor liquor fermentation. (A) Relative abundance of the active microbial community at the genus level. (B) Relative abundance of the active microbial community at the species level. DHQ, DH, and DHH represent the stages of early fermentation (0 to 6 days), middle fermentation (6 to 12 days), and late fermentation (12 to 44 days), respectively.

A total of 421 genera were annotated and the relative abundance of the top 20 predominant genera accounted for over 95% of all the microorganisms. Relative abundance of the active microbial community at the genus level is shown in Fig. 1A. Specifically, 12 fungal genera (*Saccharomyces*, Aspergillus, *Talaromyces*, *Clavispora*, *Rhizophagus*, *Cyberlindnera*, *Wickerhamomyces*, *Kluyveromyces*, *Colletotrichum*, *Jaapia*, *Mixia*, *Pichia*) and 7 bacterial genera (*Lactobacillus*, Streptococcus, *Faecalibacterium*, *Thermotoga*, *Pediococcus*, *Yersinia*, Campylobacter) constituted the 20 most abundant active microbial community. The LFL fermentation could be divided into three stages, including the stage DHQ (early fermentation, 0 to 6 days), the stage DH (middle fermentation, 6 to 12 days), and the stage DHH (late fermentation, 12 to 44 days) according to the temperature curve (Fig. S1). The genera *Lactobacillus*, Aspergillus, *Saccharomyces*, *Talaromyces*, and *Pediococcus* were the predominant microorganisms in the stage DHQ. The stage DH was marked by the rapid proliferation of Streptococcus, *Clavispora*, and *Talaromyces*, as well as the dramatic decline of *Saccharomyces*, Aspergillus, and *Pediococcus*. The stage DHH was characterized by the dramatic increment of *Faecalibacterium* and *Thermotoga*. *Lactobacillus* was the stable genus throughout the whole fermentation process. Interestingly, L. otakiensis and L. helveticus were the main species in the stage DHQ, while L. brevis and L. pasteurii were the predominant species in the stage DH. When the fermentation proceeded to the stage DHH, the abundance of L. acetotolerans increased sharply. Therefore, the different species of *Lactobacillus* in turn dominated the distinct stages of fermentation (Fig. 1B). Taken together, the active microbial community displayed an obvious succession in LFL fermentation, indicating that the fermentation possessed a specific dynamic composition.

### Dynamic changes of environmental factors.

10 environmental factors were measured during the fermentation process (Fig. S1). The ethanol production increased to the highest level (5.29 ± 0.47%) on the 44th day. The temperature reached the highest level (28.8 ± 1.06°C) on the 10th day. Acidity and moisture increased with the fermentation process, accompanied by the gradual decrease of pH and starch content. The highest production of acidity and moisture was 2.93 ± 0.12% and 66.53 ± 1.10%, respectively. The pH and starch decreased from 4.77 to 3.30 ± 0.038 and 26.70 ± 0.63% to 12.30 ± 0.20%, respectively. Reducing sugar reached the highest level (2.76 ± 0.11%) on the 4th day. Lactic acid displayed a upward trend, and its highest production was 0.26 ± 0.038%. The highest activities of glucoamylase and α-amylase were 69.67 ± 27.57 U/g and 25.12 ± 2.05 U/g on the 2nd and the 6th day, respectively.

### Environmental factors have a great impact on the succession of a microbial community.

**FIG 2:**
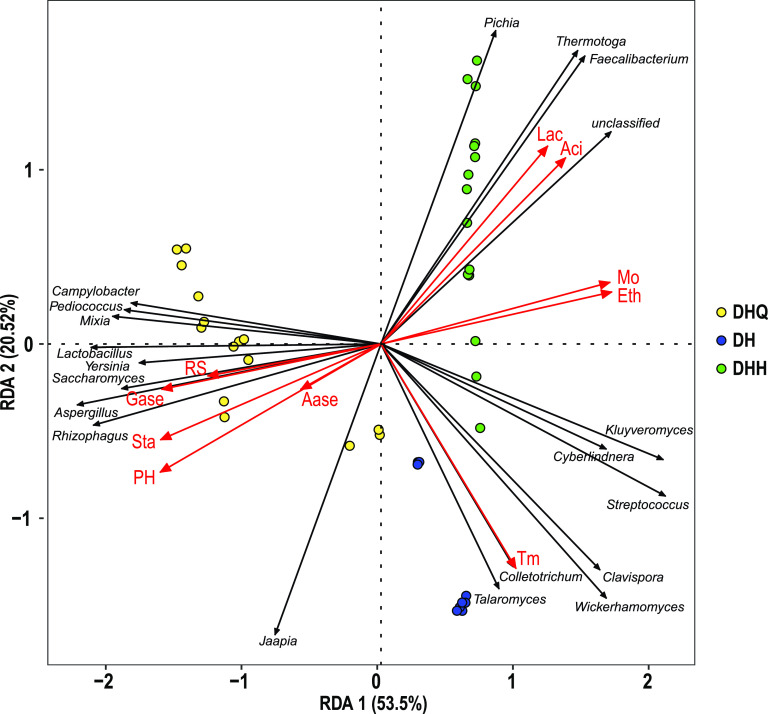
Redundancy analysis (RDA) of the active microbial community and environmental factors. The significance of variables was evaluated by the envfit test (*n* = 999). RS, reducing sugar; Sta, starch; Aci, acidity; T_m_, temperature; Eth, ethanol production; Mo, moisture; Aase, α-amylase; Gase, glucoamylase; Lac, lactic acid.

To study the correlation between the active microorganisms and the environmental factors, RDA was performed to explore the variations caused by environmental factors, and then the driving forces of the active microbial succession were suggested (Fig. 2). The explanatory rate of environmental factors on the distribution of microbial communities was 74.02%, indicating that environmental factors have a great impact on microbial succession. The factors, including pH (r^2^ = 0.97, *P* = 0.001), starch (r^2^ = 0.90, *P* = 0.001), and glucoamylase activity (r^2^ = 0.82, *P* = 0.001), especially the pH, had a strong positive correlation with the active microorganisms in the stage DHQ. In the stage DH, temperature (r^2^ = 0.75, *P* = 0.001) had a relatively high positive correlation with the active microorganism. Moisture (r^2^ = 0.91, *P* = 0.001), acidity (r^2^ = 0.89, *P* = 0.001), ethanol (r^2^ = 0.91, *P* = 0.001) and lactic acid (r^2^ = 0.83, *P* = 0.001) in the stage DHH were strong positively correlated with the microbial community. Combined with the statistical analysis of every combinational environmental factor on the contribution of the microbial community, pH, ethanol, moisture, and starch were the main driving factors for microbial succession in LFL fermentation.

### Expression profile of carbohydrate-active enzymes (CAZy) and the contributed microorganisms of the most abundant CAZy family.

**FIG 3:**
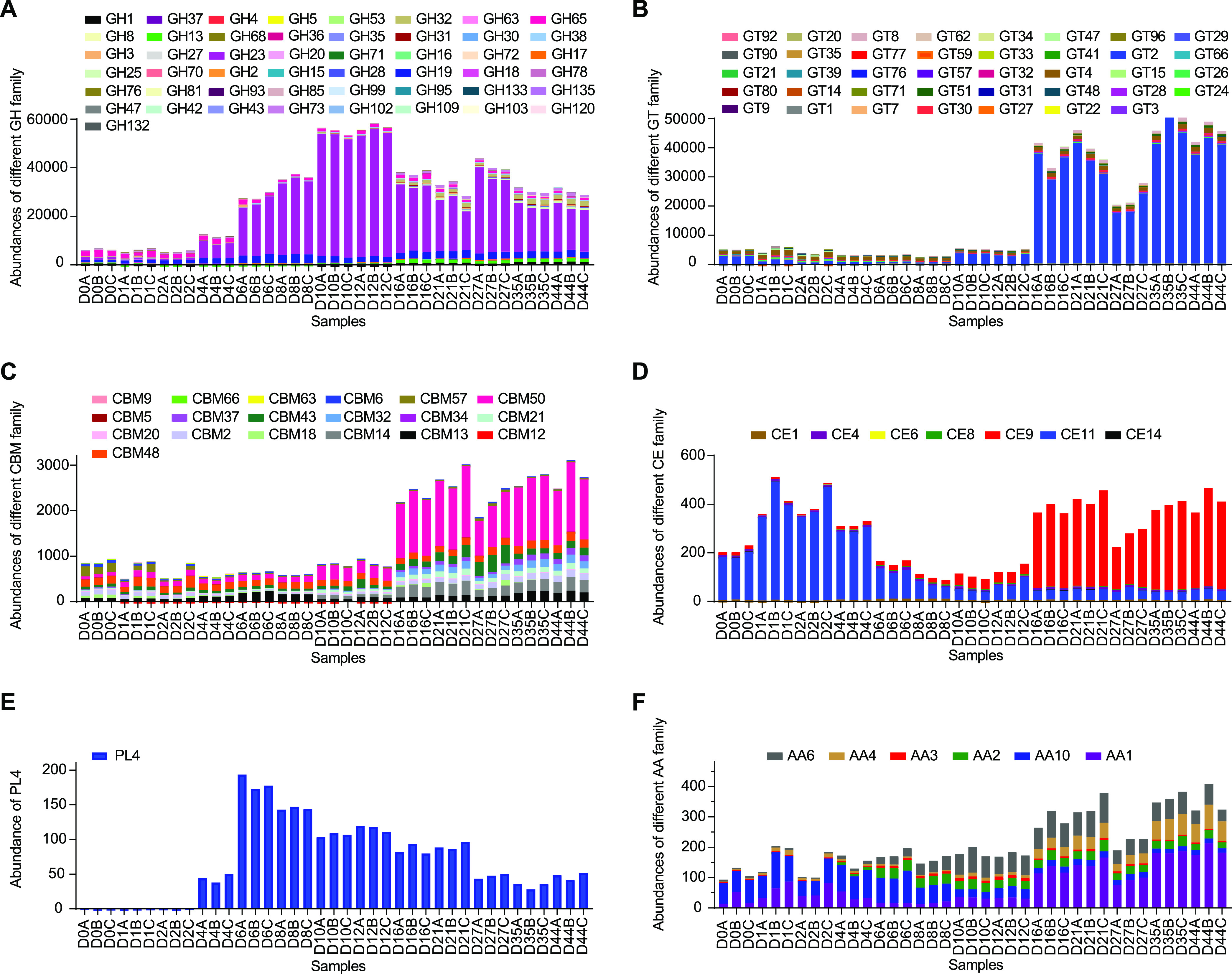
Abundances of the different carbohydrate-active enzyme families in light-flavor liquor fermentation. (A) Abundances of the glycoside hydrolases (GH) family. (B) Abundances of the glycosyltransferases (GT) family. (C) Abundances of the carbohydrate-binding modules (CBM) family. (D) Abundances of the carbohydrate esterases (CE) family. (E) Abundance of the polysaccharide lyase family 4. (F) Abundances of the auxiliary activities (AA) family. “Abundances” indicate the total abundances of the transcripts mapping to each family.

The abundance of unigenes corresponding to CAZy was calculated (Table S4) and analyzed. The results showed glycoside hydrolases (GH), glycosyltransferases (GT), carbohydrate-binding modules (CBM), carbohydrate esterases (CE), auxiliary activities (AA), and polysaccharide lyases (PL) had a descending order of the relative abundance (Fig. S2). Among them, GH23, GT2, CBM50, CE9, AA1, and PL4 had the highest abundance in their respective family (Fig. 3). GH was classified by their functions as amylase (GH13, GH15, and GH133), cellulases (GH1, GH3, and GH8), hemicellulases (GH30), and oligosaccharide-degrading enzymes (GH17, GH2, GH27, GH31, GH32, GH35, GH37. GH38, GH42, GH47, GH5, GH53, GH63, and GH99) (Fig. S3). Subsequently, the contributed microorganisms of the most abundant CAZy families were analyzed (Fig. S4). The GH23 were mainly from L. pasteurii, L. crispatus, and P. pentosaceus, and had the highest expression level in the stage DH. GT2 was mainly from F. prausnitzii and had the highest expression level in the stage DHH. The CBM50, CE9, and AA1 were mainly secreted by L. acetotolerans, and their encoding genes had the highest abundance in the stage DHH. Unlike the above most abundant CAZy families, the PL4 was from S. cerevisiae and showed comparable abundance during the three-stage fermentation.

### Carbohydrate metabolism is the key KEGG pathway.

KEGG was analyzed to reveal the highly expressed enzymes and the key pathways (Fig. S5). A total of 10,365 unigenes were annotated in the KEGG reference pathways, accounting for 28.14% of all unigenes. Annotated KEGG pathways included metabolism, genetic information processing, environmental information processing, cellular processes, and organismal systems. Metabolism was the most enriched KEGG pathway accounting for 54.64% and the carbohydrate metabolism was ranked the first (Fig. S5A). Additionally, the top 25 enriched KEGG pathways were shown in Fig. S5B. Among them, eight KEGG pathways involved in carbohydrate metabolism and glycolysis had a larger number of unigenes. Oxidative phosphorylation with a larger number of unigenes was regarded as one representative of energy metabolism. Glycerolipid metabolism and metabolism of alanine, aspartate, and glutamate were also included.

### Highly expressed enzymes of glycolysis and their contributed microorganisms.

**FIG 4:**
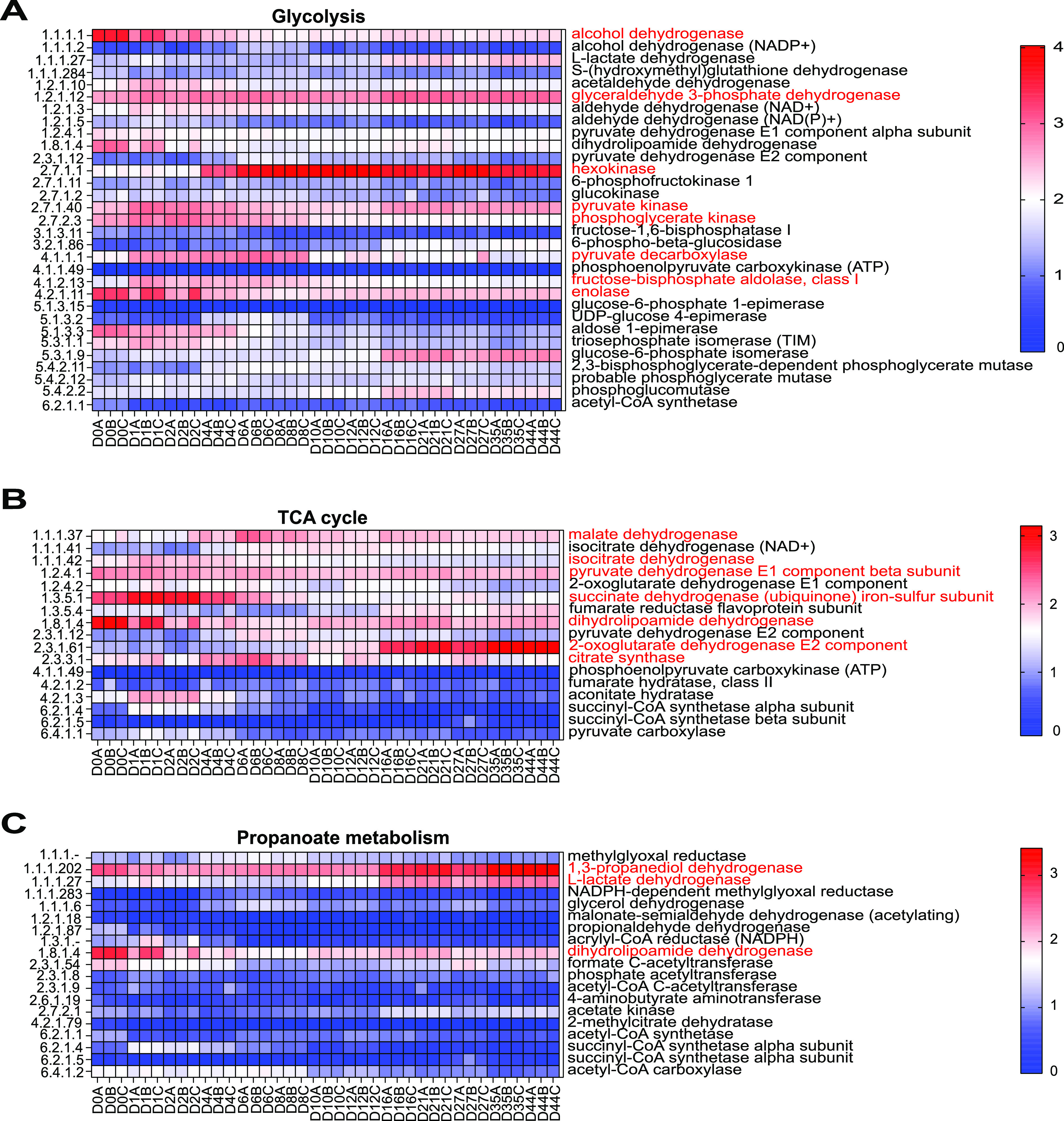
Heatmap profiles of the carbohydrate metabolic pathways, including glycolysis (A), TCA cycle (B), and propanoate metabolism (C). For all pathways, the highly expressed enzymes were marked in red. The EC number on the left side of the figure corresponds to the enzyme on the right.

Heatmap profiles and comparative analysis of glycolysis were performed. Additionally, the contributed microorganisms of the highly expressed enzymes were systematically studied (Fig. S6). A total of 31 enzymes were involved in glycolysis and eight enzymes had the higher abundance including hexokinase, pyruvate kinase, alcohol dehydrogenase, pyruvate decarboxylase, phosphoglycerate kinase, fructose-bisphosphate aldolase, glyceraldehyde 3-phosphate dehydrogenase and enolase (Fig. 4A). Hexokinase was the most abundant enzyme, and it was mainly produced by W. ciferrii. The pyruvate kinase and glyceraldehyde 3-phosphate dehydrogenase had a higher comparable expression in the stages DHQ and DHH, and they were mainly from *Lactobacillus* and *Saccharomyces*. The alcohol dehydrogenase, phosphoglycerate kinase, fructose-bisphosphate aldolase, pyruvate decarboxylase, and enolase displayed a similar expression pattern, namely, the expression gradually decreased with the fermentation process, and they were produced by bacterial and fungal members, including *Lactobacillus*, *Pediococcus*, S. cerevisiae, *Wickerhamomyces*, Naumovozyma castellii, Scheffersomyces stipites, and Ogataea parapolymorpha. Based on those data, the genera *Lactobacillus*, *Wickerhamomyces*, *Saccharomyces*, *Pediococcus*, *Naumovozyma*, *Scheffersomyces*, and *Ogataea* were the key contributors to glycolysis.

### Highly expressed enzymes of the TCA cycle and their contributed microorganisms.

A total of 17 enzymes were involved in the pathway of the TCA cycle, and seven enzymes (pyruvate dehydrogenase, citrate synthase, 2-oxoglutarate dehydrogenase, isocitrate dehydrogenase, dihydrolipoamide dehydrogenase, succinate dehydrogenase, and malate dehydrogenase) with higher abundance were chosen for further analysis (Fig. 4B). The 2-oxoglutarate dehydrogenase had the highest expression level in the stage DHH and it was mainly from W. ciferrii. The succinate dehydrogenase, dihydrolipoamide dehydrogenase, isocitrate dehydrogenase, and citrate synthase displayed a similar expression pattern, namely, having a higher expression level in the stage DHQ, and then dramatically decreasing in the stages DH and DHH. The contributed microorganisms were C. tropicalis, S. cerevisiae, Escherichia coli, and *Lactobacillus*. The malate dehydrogenase and pyruvate dehydrogenase had a higher comparable expression in the stages DH and DHH, and their contributed members included C. fabiani and *Lactobacillus* (Fig. S7). Generally, the genera *Lactobacillus*, *Wickerhamomyces*, *Saccharomyces*, *Candida*, Escherichia, and *Cyberlindnera* were the key microbial members in the pathway of the TCA cycle.

### Highly expressed enzymes of propanoate metabolism and their contributed microorganisms.

A total of 19 enzymes were involved in the propanoate metabolism. Of them, three highly expressed enzymes, including l-lactate dehydrogenase, 1,3-propanediol dehydrogenase, and dihydrolipoamide dehydrogenase were further analyzed (Fig. 4C). l-lactate dehydrogenase and 1,3-propanediol dehydrogenase displayed the highest expression level in the stage DHH and were mainly produced by *Lactobacillus* (Fig. S8). Dihydrolipoamide dehydrogenase was also involved in the pathway of the TCA cycle. Therefore, *Lactobacillus* was the main contributing microorganism in the pathway of propanoate metabolism.

### Highly expressed enzymes of oxidative phosphorylation and their contributed microorganisms.

**FIG 5:**
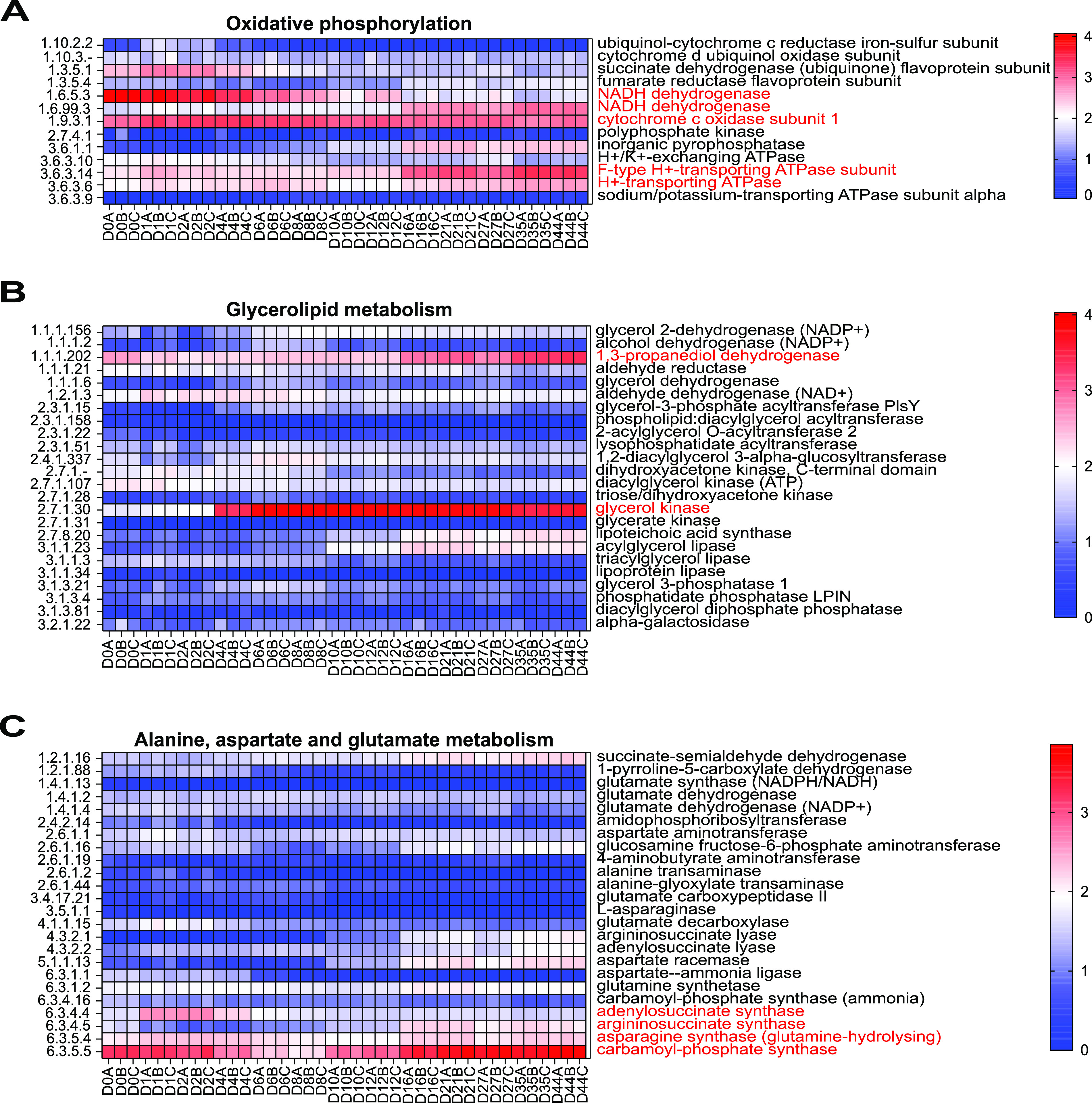
Heatmap profiles of the pathways involved in oxidative phosphorylation (A), the glycerolipid metabolism (B), and alanine, aspartate, and glutamate metabolism (C). For all pathways, the highly expressed enzymes were marked in red. The EC number on the left side of the figure corresponds to the enzyme on the right.

Thirteen enzymes involved in oxidative phosphorylation were identified. Of them, four enzymes, including the cytochrome C oxidase subunit, the NADH dehydrogenase, the H+-transporting ATPase, and the F-type H+-transporting ATPase subunit displayed a relatively high expression level (Fig. 5A). The cytochrome c oxidase subunit and NADH dehydrogenase (EC1.6.5.3) had a higher expression level in the stage DHQ and then decreased in the stages DH and DHH, and they were mainly produced by the genera *Saccharomyces* and *Lichtheimia*. The H+-transporting ATPase (EC3.6.3.6 and EC3.6.3.14) and NADH dehydrogenase (EC1.6.99.3) had a higher expression level in the stage DHH and were produced by a large variety of microorganisms, including *Lactobacillus*, *Saccharomyces*, and *Candida* (Fig. S9). Overall, the genera *Lactobacillus*, *Saccharomyces*, *Candida*, and *Lichtheimia* were the main active microorganisms in the pathway of oxidative phosphorylation.

### Highly expressed enzymes of glycerolipid metabolism, alanine, aspartate, and glutamate metabolism, and their contributed microorganisms.

Twenty-four enzymes were found in the pathway of glycerolipid metabolism. Of them, glycerol kinase and 1,3-propanediol dehydrogenase were highly expressed (Fig. 5B). The abundance of these two enzymes was increased at the late stage of fermentation (Fig. S10). Unexpectedly, the genus *Lactobacillus* was almost the only contributing microorganism in the pathway of glycerolipid metabolism.

Twenty-four enzymes were involved in the pathway of alanine, aspartate, and glutamate metabolism (Fig. 5C). The abundance of argininosuccinate synthase and carbamoyl-phosphate synthase was kept at a stable level in the stages DHQ and DH and then increased significantly in the stage DHH. They were mainly from *Lactobacillus*, P. pentosaceus, Absidia idahoensis, and S. cerevisiae. Moreover, the abundance and the contributed microorganisms of adenylosuccinate synthase and asparagine synthase were also analyzed. The results demonstrated that the genera *Lactobacillus*, *Saccharomyces*, and *Wickerhamomyces* were the main contributors (Fig. S11). Generally, the genera *Lactobacillus*, *Saccharomyces*, *Wickerhamomyces*, *Pediococcus*, and *Absidia* were the most active microorganisms in the pathway of alanine, aspartate, and glutamate metabolism. Furthermore, all enzymes in the above pathways except for the oxidative phosphorylation were comprehensively analyzed. The location and abundances of all enzymes in their respective pathways are shown in Fig. S12 to 16, respectively.

### Functional model of carbohydrate hydrolysis, ethanol production, and flavor generation.

**FIG 6:**
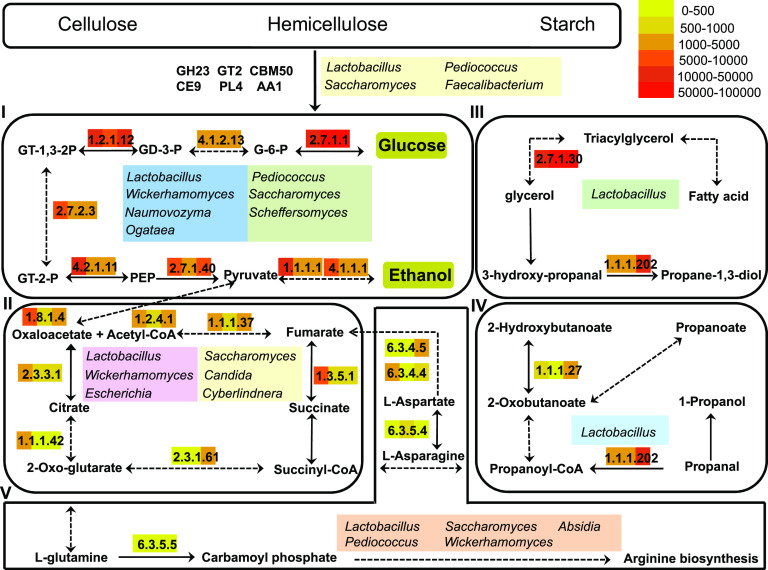
Functional model of carbohydrate hydrolysis, ethanol production, and flavor generation in light-flavor liquor fermentation. The highly expressed enzymes and the contributed microorganisms were listed. I, II, III, IV, and V represent the pathways of glycolysis, TCA cycle, glycerolipid metabolism, propanoate metabolism, and alanine, aspartate, and glutamate metabolism, respectively. G-6-P, glucose-6-phosphate; GD-3-P, glyceraldehyde-3-phosphate; GT-1,3-2P, glycerate-1,3-phosphate; GT-2-P, glycerate-2-phosphate; PEP, phosphoenolpyruvate. EC2.7.1.1, hexokinase; EC2.7.1.40, pyruvate kinase; EC1.1.1.1, alcohol dehydrogenase; EC4.1.1.1, pyruvate decarboxylase; EC1.2.1.12, glyceraldehyde 3-phosphate dehydrogenase; EC2.7.2.3, phosphoglycerate kinase; EC4.1.2.13, fructose-bisphosphate aldolase; EC4.2.1.11, enolase; EC1.2.4.1, pyruvate dehydrogenase; EC2.3.3.1, citrate synthase; EC2.3.1.61, 2-oxoglutarate dehydrogenase; EC1.1.1.42, isocitrate dehydrogenase; EC1.3.5.1, succinate dehydrogenase flavoprotein subunit; EC1.8.1.4, dihydrolipoamide dehydrogenase; EC1.1.1.37, malate dehydrogenase; EC1.1.1.202, 1,3-propanediol dehydrogenase; EC1.1.1.27, l-lactate dehydrogenase;EC2.7.1.30, glycerol kinase; EC6.3.4.4, adenylosuccinate synthase; EC6.3.4.5, argininosuccinate synthase; EC6.3.5.4, asparagine synthase; EC6.3.5.5, carbamoyl-phosphate synthase. Black solid lines represent the one-step reaction. Black dashed lines represent the multiple-step reaction. The key enzymes are highlighted with color. From left to right, three parts of color bars represent the stages of early fermentation (0 to 6 days), middle fermentation (6 to 12 days), and late fermentation (12 to 44 days), respectively. The color indicates the enzyme expression level based on the abundance of unigenes.

Based on the metatranscriptomic analysis, one model of the carbohydrate hydrolysis, ethanol production, and flavor generation in LFL fermentation were proposed in Fig. 6. The raw materials used here included barley, wheat, sorghum, and peas and their main ingredients were starch, cellulose, and hemicellulose. Carbohydrate hydrolysis required a combination of multiple CAZy responsible for converting polymers to monomers. The key CAZy (GH23, GT2, PL4, CE9, AA1, and CBM50) were mainly produced by *Lactobacillus*, *Pediococcus*, *Saccharomyces*, and *Faecalibacterium*. Subsequently, the monomers were further utilized by the metabolic pathways. Glycolysis is related to alcohol production and eight highly expressed enzymes were mainly produced by *Lactobacillus*, *Pediococcus*, *Wickerhamomyces*, *Saccharomyces*, *Ogataea*, *Naumovozyma*, and *Scheffersomyces*. A proper combination of the active microorganisms would improve alcohol production. There was a close relationship between the pathway of the TCA cycle and the flavor generation. The key reaction steps and enzymes were marked in the TCA cycle. These key enzymes were mainly from the genera *Lactobacillus*, *Wickerhamomyces*, *Saccharomyces*, *Candida*, *Cyberlindnera*, and Escherichia. The pathways of propanoate metabolism and glycerolipid metabolism were mainly contributed to flavor generation. Unexpectedly, the key enzymes were mainly from the genus *Lactobacillus*. The generation of the higher alcohols is mainly from amino acid metabolism, which contributed to the flavor generation by the formation of α-ketoacids and corresponding alcohols successively (18). There were four highly expressed enzymes, which were mainly produced by *Lactobacillus*, *Pediococcus*, *Wickerhamomyces*, *Saccharomyces*, and *Absidia* in the pathway of alanine, aspartate, and glutamate metabolism. Based on the contributors of key CAZy and the important metabolic pathways, the core active microorganisms were proposed as *Saccharomyces*, *Lactobacillus*, *Wickerhamomyces*, *Pediococcus*, *Candida*, and *Faecalibacterium* in LFL fermentation. In general, many active microorganisms interacted with each other and produced various key enzymes, finally promoting carbohydrate hydrolysis, ethanol production, and flavor generation.

## DISCUSSION

In this study, metatranscriptomic analysis was applied to reveal the core and functional active microbial community in LFL fermentation. By analyzing the 20 most abundant genera, *Faecalibacterium* was the predominant microorganism in the stage DHH. However, there are no reports about the roles of *Faecalibacterium* in liquor fermentation except *Faecalibacterium* was also detected as one dominant member (19). Previous studies reported that *Faecalibacterium* as a potential producer of hemicellulase is involved in the butyrate metabolism in the human body (20, 21). That GT2 as the most abundant GT family was mainly from F. prausnitzii proved its possible functions. Structures of the microbial community from the DNA-based and RNA-based sequencing are quite different, indicating some microorganisms with high abundance may not play the important functions (22, 23). Therefore, metatranscriptomic analysis helps us to find the active microorganisms and their novel functions.

The composition and diversity of microbial communities are the basis of flavor generation. For SFL fermentation, alcohols (n-propanol and iso-butanol), acids (caproic acid, lactic acid, acetic acid, and butyric acid), and their corresponding ethyl esters are the main flavor compounds (24, 25). *Clostridium* and *Bacillus* are responsible for the production of caproic acid and butyric acid (26–28). Lactic acid bacteria (LAB) and *Acetobacter* mainly produce lactic acid and acetic acid, respectively. Ethyl ester is mainly produced by *Penicillium*, Aspergillus, *Emericella*, *Rhizopus*, *Cladosporium*, *Mucor*, *Hansenula*, *Candida*, etc (24). Ethyl acetate, the main flavor compound in LFL fermentation, is mainly produced by *Wickerhamomyces* (19). Additionally, terpenoids (geraniol, α-terpineol, linalool, etc), as the trace light-flavor compounds, are mainly produced by S. cerevisiae, P. kudriavzevii, and W. anomalus (29). The composition of flavor compounds is the most complex in JFL fermentation. Bacterial genera were relatively richer than those in LFL or SFL fermentation, and they included *Bacillus*, *Lactobacillus*, *Acetobacter*, *Clostridium*, *Oceanobacillus*, *Thermoactinomyces*, *Virgibacillus*, *Pediococcus*, *Kroppenstedtia*, etc (30, 31). Among them, *Bacillus* are responsible for the production of characteristic compounds pyrazines, propanoic acid, 1,3-butanediol, acetate, methyl ester, etc (32). The predominant fungal genera were Aspergillus, *Trichoderma*, *Rhizopus*, *Mucor*, *Monascus*, *Pichia*, *Candida*, *Torulaspora*, *Saccharomyces*, *Hansenula*, *Cyberlindera*, *Schizosaccharomyces*, Zygosaccharomyces, *Trichosporon*, etc. (31, 33). Generally, *Saccharomyces*, especially S. cerevisiae, produce alcohol, while other yeasts are responsible for producing main flavor compounds, such as acids, higher alcohols, aldehydes, ketones, esters, and terpenes (34, 35). Additionally, the bacteria are responsible for saccharification, protein degradation, and flavor generation during fermentation (36–38). Microbial community composition is quite complicated, and that of the same flavor type in different producing regions changes significantly. Although great contributions have been made, more functional microorganisms need to be found in the future.

Reconstruction of metabolic pathways provides the basis for a deep understanding of the fermentation features and further improves the qualities of the fermented foods (39). Many successful examples of the application of *Wickerhamomyces* to improve the liquor flavors were reported (19, 40, 41). Owing to the application, one high ethyl acetate producing strain *Wickerhamomyces* sp. was isolated from our samples, and then reinoculated in the fermented grains. Interestingly, the abundance of the ethyl acetate increased, and the quality of the liquor has been significantly improved (Y. Wang and W. Hao, unpublished data). The contribution of *Lactobacillus* is great during fermentation. However, the high contents of ethyl lactate caused by *Lactobacillus* also are a disadvantage for fermentation. Bioaugmentation of *Bacillus* isolated from the Daqu reduced the abundance of lactic acid and balanced the ratios between ethyl acetate and ethyl lactate (Y. Wang and W. Hao, unpublished data).

Liquor brewing is an open solid-state fermentation process, and the composition of the active microorganisms was determined by multiple parameters, such as sampling regions, seasons, fermented containers, producing processes, raw materials, Daqu, etc (42). Therefore, the results in this study only were representative of this batch fermentation. To better understand the composition and functions of the active microorganisms, more representative sampling is needed. Additionally, there are some limitations in RNA and bioinformatics analysis. Easily degradation of mRNA for metatranscriptomic sequencing could lead to the loss of the functional genes or important metabolic pathways (10). Moreover, a large amount of fungal genetic information cannot be well-annotated due to the lack of a powerful fungal genomic database. Technological innovations and improvements in fungal genomic databases will provide an important basis for metatranscriptomic studies in the future.

LFL fermentation is controlled by active microorganisms, metabolites, and environmental factors. Here, we revealed the core active microbial community in the three-stage fermentation, confirmed the driving forces of the microbial succession, identified the most abundant saccharifying enzymes, and explored the highly expressed enzymes and the contributed microorganisms in the key KEGG pathways by metatranscriptomic analysis. It is one systematic report about the compositional dynamics, succession, and functions of the active microorganisms.

## MATERIALS AND METHODS

### Sample collection.

All samples were collected from the Niulanshan distillery (Beijing, China) in November 2019. Samples were collected in duplicate, one was used for total RNA isolation to perform the metatranscriptomic sequencing, and the other was used for measuring the fermentation parameters. One sample from each of three different pits was collected at every time point. The pits A, B, and C were sampled at 0, 1, 4, 8, 12, 21, and 35 days of fermentation, respectively. The pits D, E, and F were sampled at 2, 6, 10, 16, 27, and 44 days of fermentation, respectively. Pit G was used to fill the space left above pits after sampling. In total, 39 samples from 13 time points were collected. To ensure consistency, all samples were harvested from the locations about 40 to 50 cm away from the ground using a homemade cylindrical tool and mixed well to form one sample (Fig. S17). D0A, D0B, and D0C represent the samples isolated from the initial fermentation in pits A, B, and C, respectively. D2A, D2B, and D2C represent the samples isolated from the 2nd day of fermentation in pits D, E, and F, respectively. All samples were named in the same way.

### RNA extraction.

Microbial isolation was performed as described previously (16). 2 g of isolated cells were ground into the fine powder with liquid nitrogen. Then, 0.1 g of cells were mixed with 600 μL of TES buffer (0.3 M sucrose, 25 mM Tris-HCl (pH 8.0), 25 mM EDTA (pH 8.0), autoclaved for use), and 600 μL acidic phenol at 65°C for 10 min. The lysate was centrifuged, and the supernatant was mixed thoroughly with 200 μL acidic phenol-chloroform/isoamylol (25:24:1) and chloroform/isoamylol (24:1) successively. The RNA was treated with DNase 1 (Promega, USA) according to the manufacturer’s protocol. The purity and concentration of total RNA were analyzed using Bioanalyzer 2100 and RNA 1000 Nano Lab Chip kit (Agilent, CA, USA) with RIN number >7.0.

### cDNA library construction and metatranscriptomic sequencing.

An approximate 5 μg of total RNA was used to deplete rRNA. The remaining RNA was fragmented into small pieces by divalent cations under higher temperatures. Then mRNA was reverse transcribed to cDNA. The cDNA was next used to synthesize U-labeled second-stranded DNA with E. coli DNA polymerase I, RNase H, and dUTP. A-base was then added to the blunt ends of each strand for ligation with the adapters with T-base. Dual-index adapters were ligated to the fragments, and size selection was performed with AMPureXP beads. After U-labeled second-stranded DNA was treated with the heat-labile UDG enzyme, the ligated products were amplified with PCR by the following conditions: denaturation at 95°C for 3 min; 8 cycles of denaturation at 98°C for 15 sec, annealing at 60°C for 15 sec, and extension at 72°C for 30 sec; and final extension at 72°C for 5 min. The average insert size for the paired-end library was 300 bp. Finally, paired-end sequencing was performed on an IlluminaHiseq 4000 at the LC Sciences (Hangzhou, China).

### Data analysis.

When the qualified reads from the raw data were obtained, they were *de novo* assembled by Trinity v2.4.0. All contigs were clustered by CD-HIT v4.6.1 to obtain unigenes. The abundance of unigenes was calculated by TPM based on the numbers of aligned reads by bowtie2 v2.2.0. The lowest common ancestor taxonomy of unigenes was obtained by aligning them against the NCBI NR database by DIAMOND v0.7.12. Similarly, the functional annotation of unigenes was obtained. The metatranscriptomic database has been submitted to NCBI SRA and is accessible under PRJNA773513.

### Determination and analysis of fermentation parameters.

The temperature was monitored and recorded by a thermograph. Moisture was determined using a dry/wet method. The pH was measured by a pH meter. The acidity was detected by titration with 0.1 M NaOH solution until reached an endpoint of pH 8.2. Ethanol was analyzed by the high-performance liquid chromatography (HPLC, Waters 2695, Sunfire C18 [150 mm × 4.6 mm, 5 μm]) equipped with a photodiode array detector. The samples (5 g) were mixed with the extraction buffer (10 mL), and treated with ultrasonic, and then the sample solution was centrifuged and filtered for HPLC analysis. The HPLC condition was as follows: the mobile phase was water and acetone (96:4), and the elution was performed at 0.8 mL/min in 4 min. The detection wavelength was 233 nm. Reducing sugar was detected by the ultrahigh-performance liquid chromatography (UPLC, ACQUITY UPLC BEH C18 [100 mm × 2.1 mm, 1.7 μm], Waters) equipped with an evaporative light-scattering detector. The samples (3 g) were mixed with 70% acetonitrile (10 mL) and treated with ultrasonic, the sample solution was centrifuged and filtered for UPLC analysis. The UPLC condition was as follows: the mobile phase was water and acetonitrile with 0.2% triethylamine, and UPLC was done at 0.15 mL/min using gradient elution from 85% to 78% acetonitrile in the first 2 min, and then from 78% to 50% acetonitrile in the following 9 min. Lactic acid was also detected by UPLC. Five grams of the samples were mixed with 5 mL of water, and then the sample solution was centrifuged and filtered for UPLC analysis. The UPLC condition was as follows: the mobile phase was water and 2.5 mM ammonium dihydrogen phosphate (95:5), and UPLC was done at 0.2 mL/min in 10 min. The detection wavelength was 210 nm. The activity of α-amylase was measured according to the recommendation of the amylase assay kit (Beijing Solarbio Science & Technology Co., Ltd., China). One unit of α-amylase was defined as the amount of enzyme that catalyzes 1 g of samples to obtain 1 mg of reducing sugar per minute. Starch and glucoamylase activity was determined according to the national professional standard methods GB5009.9-2016 and GB1886.174-2016, respectively. One unit of glucoamylase activity was defined as the amount of enzyme that catalyzes 1 g of samples to release 1 mg of glucose per hour from soluble starch in sodium acetate buffer (50 mM, pH 4.6) at 40°C.

### Statistical analysis.

Composition of the active microbial community, determination of fermentation parameters, abundances of CAZy, and key metabolic pathways were conducted by Excel 2019. RDA was performed in R (version 3.6.3) via the vegan package (version 2.5-7). The RDA figure was generated using the OmicStudio tools at https://www.omicstudio.cn/tool. The envfit test is conducted to determine the correlation between the predominant genera and environmental factors in R (version 3.6.3) via the vegan package (version 2.5-7). The values of r^2^ (coefficient of determination) quantify the goodness of fit. A higher value indicates the higher impact of the environmental factor on microorganism distribution. The significant correlations (*P* < 0.05) were chosen.

### Data availability.

The data sets presented in this work can be found in the repository NCBI SRA under accession number (PRJNA773513).
