# Supplementary material for: Metatranscriptomics Unravel Composition, Drivers, and Functions of the Active Microorganisms in Light-Flavor Liquor Fermentation
Source: Microbiol Spectr. 2022 May 31;10(3):e02151-21. doi: 10.1128/spectrum.02151-21 (PMC9241730; doi:10.1128/spectrum.02151-21)
Supplement: SUPPLEMENTAL FILE 1 — Supplemental material. Download spectrum.02151-21-s0001.pdf, PDF file, 1.5 MB [file spectrum.02151-21-s0001.pdf]

SUPPLEMENTARY MATERIALS

Metatranscriptomics Unravel Composition, Drivers and Functions of the Active Microorganisms in Light-Flavor Liquor Fermentation

Yuanyuan Pan[a,#], Ying Wang[b,#], Wenjun Hao[b], Chengbao Duan[a,c], Shiyuan Wang[a,c], Jinwang Wei[b,]* and Gang Liu[a,c,]*

a, State Key Laboratory of Mycology, Institute of Microbiology, Chinese Academy of Sciences, Beijing 100101, China

b, Beijing Shunxin Agriculture Company Limited, Niulanshan Distillery, Beijing 101301, China

c, University of Chinese Academy of Sciences, Beijing 100049, China

Running Head: Metatranscriptomic Analysis in Liquor Fermentation

*Address correspondence to Jinwang Wei, weijinwang1971@163.com, or Gang Liu, liug@im.ac.cn.

#Yuanyuan Pan and Ying Wang contributed equally to this work. Author order was determined on the basis of seniority.

Supplementary Tables

Table S1 Raw and clean data statistics of Light-flavor liquor samples by metatranscriptomic sequencing. D0A, D0B and D0C represent the samples isolated from the initial fermentation in pits A, B and C, respectively. D2A, D2B and D2C represent the samples isolated from the 2nd day of fermentation in pits D, E and F, respectively. All samples were named in the same way.

Samples	Raw Data		Valid Data		Valid%	Q20%	Q30%	GC%
	Read	Base	Read	Base				
D0A	62635418	8.83G	62151584	8.66G	99.23	98.32	95.19	45.14
D0B	64359858	9.07G	63924458	8.91G	99.32	98.38	95.32	44.94
D0C	71328302	10.06G	70702554	9.85G	99.12	98.30	95.16	44.90
D1A	83372364	11.76G	81486540	11.35G	97.74	98.21	94.41	44.96
D1B	75154684	10.60G	73843510	10.29G	98.26	98.21	94.36	44.26
D1C	64258534	9.06G	62913196	8.76G	97.91	98.38	94.81	45.06
D2A	86033078	12.13G	84480712	11.77G	98.20	98.34	94.71	45.15
D2B	86510734	12.20G	84885582	11.82G	98.12	98.28	94.58	45.27
D2C	75097116	10.59G	73886688	10.29G	98.39	98.31	94.64	44.80
D4A	82522764	11.64G	81030310	11.28G	98.19	98.33	94.72	45.88
D4B	84662044	11.94G	82988896	11.55G	98.02	98.31	94.71	45.88
D4C	99732220	14.06G	97834220	13.62G	98.10	98.25	94.54	45.66
D6A	99524740	14.03G	97396994	13.56G	97.86	98.22	94.56	47.53
D6B	64119722	9.04G	62756118	8.76G	97.87	98.38	94.88	47.52
D6C	62220628	8.77G	61157272	8.53G	98.29	98.31	94.71	47.25
D8A	69661664	9.82G	68265868	9.52G	98.00	98.27	94.66	48.17
D8B	59311164	8.36G	58257430	8.13G	98.22	98.31	94.73	48.05
D8C	78379164	11.05G	76923692	10.73G	98.14	98.31	94.72	48.14
D10A	61890710	8.73G	61082880	8.53G	98.69	98.55	95.32	47.82
D10B	73647500	10.38G	72335124	10.09G	98.22	98.26	94.59	47.74
D10C	78966408	11.13G	77540812	10.81G	98.19	98.26	94.60	47.96
D12A	62590176	8.83G	61618674	8.60G	98.45	98.44	94.98	47.68
D12B	64569032	9.10G	63488400	8.86G	98.33	98.34	94.77	47.61
D12C	69089702	9.74G	67906354	9.47G	98.29	98.31	94.70	47.75
D16A	60881030	8.58G	59951522	8.36G	98.47	98.33	94.70	44.79
D16B	72897964	10.28G	71834646	10.02G	98.54	98.34	94.71	44.58
D16C	67479500	9.51G	66536874	9.28G	98.60	98.48	95.09	44.87
D21A	68139832	9.61G	67026914	9.34G	98.37	98.26	94.51	44.00
D21B	67241882	9.48G	66178506	9.23G	98.42	98.24	94.48	44.25
D21C	59414288	8.38G	58581058	8.18G	98.60	98.39	94.81	43.91
D27A	57441018	8.10G	56566328	7.90G	98.48	98.33	94.68	45.89
D27B	62577624	8.82G	61810436	8.63G	98.77	98.47	95.02	45.57

D27C	58694458	8.28G	57897686	8.08G	98.64	98.32	94.64	45.11
D35A	74000726	10.43G	72829322	10.15G	98.42	98.30	94.59	43.90
D35B	56694870	7.99G	55819444	7.78G	98.46	98.27	94.53	43.36
D35C	64013106	9.03G	63036562	8.79G	98.47	98.22	94.39	43.31
D44A	61006934	8.60G	59927608	8.35G	98.23	98.21	94.37	43.92
D44B	63173936	8.91G	62371982	8.71G	98.73	98.30	94.54	42.79
D44C	78490876	11.07G	76953146	10.73G	98.04	98.23	94.39	43.31

Table S2 Assembly quality statistics of Light-flavor liquor samples by metatranscriptomic sequencing. D0A, D0B and D0C represent the samples isolated from the initial fermentation in pits A, B and C, respectively. D2A, D2B and D2C represent the samples isolated from the 2nd day of fermentation in pits D, E and F, respectively. All samples were named in the same way. Contigs represents the total numbers of contigs via assembly. Quality assessment tool for assembly is from the Center for Algorithmic Biotechnology. All statistics are based on the contigs (size ≥ 500 bp).

Samples	Contigs	Total length	Largest contig	GC (%)	N50	L50
D0A	12067	14376215	12796	40.03	1404	3092
D0B	13158	16355515	11287	40.00	1501	3352
D0C	14639	18369049	8671	40.00	1580	3684
D1A	8454	7713610	6262	39.63	939	2656
D1B	13751	17277748	9750	39.46	1512	3716
D1C	12078	12405415	7849	40.22	1115	3604
D2A	8092	7428651	5686	39.44	954	2533
D2B	8198	7545592	7857	39.48	949	2551
D2C	12380	14849576	9140	39.61	1413	3400
D4A	9946	8573201	5882	40.01	870	3275
D4B	9499	8229415	5876	40.13	878	3098
D4C	12336	11071587	6806	39.81	917	3911
D6A	11121	9753307	6451	40.65	892	3637
D6B	8741	7712843	6324	40.65	899	2823
D6C	8708	7787721	6061	40.41	919	2813
D8A	8889	7951826	7036	40.58	916	2858
D8B	8535	7648483	7233	40.43	920	2732
D8C	10482	9517080	6526	40.50	932	3338
D10A	12532	14018669	12447	41.73	1229	3077
D10B	12730	14884428	17631	40.73	1306	3044
D10C	11678	13279685	18728	40.76	1260	2836
D12A	12286	14784293	19547	40.12	1386	2949
D12B	12624	15344996	19513	40.14	1402	3055
D12C	12315	14840804	20033	40.18	1388	2952
D16A	8077	10167128	26044	40.30	1398	1535
D16B	10730	13259391	44435	40.53	1362	2154
D16C	10443	12146717	32875	41.35	1247	2249
D21A	8661	11113325	40762	40.24	1436	1541
D21B	8753	11118415	32585	40.29	1428	1669
D21C	8783	10949403	31606	40.62	1345	1663

D27A	8160	10520729	27883	39.89	1485	1551
D27B	10173	12581407	45230	40.63	1397	2043
D27C	8495	11060133	26488	39.95	1493	1568
D35A	6945	9097053	32726	40.01	1485	1162
D35B	5291	7541582	30579	39.90	1771	727
D35C	6499	9135427	54864	39.99	1672	942
D44A	7001	9248388	43898	39.98	1508	1170
D44B	8041	10466234	40768	40.01	1466	1345
D44C	7831	10499179	52438	39.61	1540	1231

Table S3 Unigenes statistics of Light-flavor liquor samples by metatranscriptomic sequencing. Integrity:start represents the numbers of unigenes containing the start codons; Integrity:end represents the numbers of unigenes containing the stop codons; Integrity:all represents the numbers of unigenes containing both the start and stop codons; Integrity:none represents the numbers of unigenes containing no any start or stop codons.

Unigenes	Features
Unigenes Numbers	36834
Integrity:start	3482 (9.45%)
Integrity:end	2414 (6.55%)
Integrity:all	286 (0.78%)
Integrity:none	30652 (83.22%)
Total Length (Mbp)	48.05
Average Length (bp)	1304.37
GC percent	40.37%

Table S4 Abundances of the carbohydrate-active enzymes in Light-flavor liquor fermentation. D0A, D0B and D0C represent the samples isolated from the initial fermentation in pits A, B and C, respectively. D2A, D2B and D2C represent the samples isolated from the 2nd day of fermentation in pits D, E and F, respectively. All samples were named in the same way. GH, glycoside hydrolases; GT, glycosyltransferases; CBM, carbohydrate-binding modules; CE, carbohydrate esterases; PL, polysaccharide lyases; AA, auxiliary activities.

Samples	GH	GT	CBM	CE	AA	PL
D0A	6110.29	5080.51	854.87	204.88	93.34	1.18
D0B	6721.28	5017.86	848.58	204.92	132.29	0.53
D0C	6278.05	5251.64	945.19	230.50	104.48	0.71
D1A	5266.43	3975.60	504.02	360.98	118.76	0.43
D1B	6195.47	6180.88	862.01	511.99	204.54	0.86
D1C	6996.57	6057.58	891.34	414.81	197.47	0.99
D2A	5229.87	3886.94	516.07	359.35	102.24	0.89
D2B	5300.83	3498.12	518.69	380.69	100.32	0.66
D2C	5848.70	5262.76	858.16	487.22	184.77	1.17
D4A	12695.25	3275.08	570.55	311.18	172.75	44.14
D4B	11252.87	3058.56	538.98	311.11	129.88	37.96
D4C	11702.39	3316.47	616.59	331.20	155.24	50.15
D6A	27383.12	3184.17	651.28	166.48	168.32	193.47
D6B	27245.04	3246.75	642.08	150.94	170.03	172.79
D6C	29941.68	3522.84	671.89	169.34	197.45	177.57
D8A	35222.12	2593.57	585.01	113.79	146.06	142.91
D8B	37478.14	2769.20	581.85	97.20	155.25	146.91
D8C	36125.14	2735.18	601.73	88.57	170.49	144.30
D10A	56467.36	5498.22	821.01	114.89	178.39	103.42
D10B	55662.06	5133.17	841.65	102.21	201.48	109.04
D10C	53537.57	5173.66	786.12	92.08	170.08	106.70
D12A	55707.38	4852.57	947.85	120.54	168.93	119.42
D12B	58307.81	4784.46	826.91	121.19	184.10	117.85
D12C	56601.73	5290.88	773.56	155.48	172.88	110.77
D16A	38037.79	41581.45	2189.27	366.26	264.06	81.58
D16B	37040.32	32990.90	2477.39	400.65	320.01	93.60
D16C	38910.04	40375.97	2272.77	362.63	278.58	79.75
D21A	32840.51	46102.07	2686.61	420.92	315.01	88.54
D21B	34436.34	39706.28	2535.68	401.69	318.41	86.39
D21C	28432.27	35956.11	3017.86	457.19	378.80	96.43
D27A	43851.24	20464.33	1861.36	223.39	189.98	43.34
D27B	39826.71	21184.19	2197.09	280.49	227.62	47.64
D27C	39221.22	27929.44	2500.52	298.68	226.45	50.23

D35A	31867.70	45847.65	2543.01	375.92	347.22	35.78
D35B	29989.55	57072.40	2754.46	396.98	359.04	28.04
D35C	29533.98	50039.29	2797.99	413.00	382.53	35.84
D44A	31768.37	41924.17	2485.90	366.41	320.85	48.34
D44B	29899.91	48996.97	3111.70	467.21	407.80	41.89
D44C	28797.89	45749.95	2736.24	411.40	324.15	51.69

Supplementary Figures

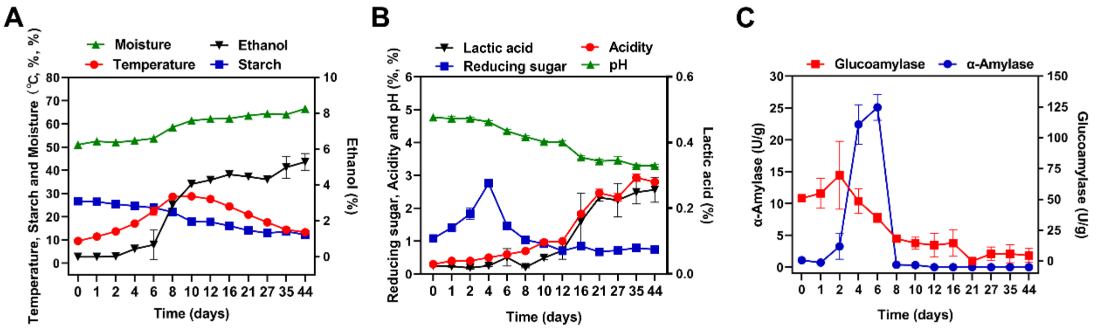

FIG S1 Profiles of the environmental factors in Light-flavor liquor fermentation. (A) Changes of temperature, moisture, ethanol and starch; (B) Changes of reducing sugar, acidity, pH and lactic acid; (C) Changes of α-amylase and glucoamylase activities. Three biologically independent samples were collected and measured, error bars = SD. Data are presented as mean values ± SD.

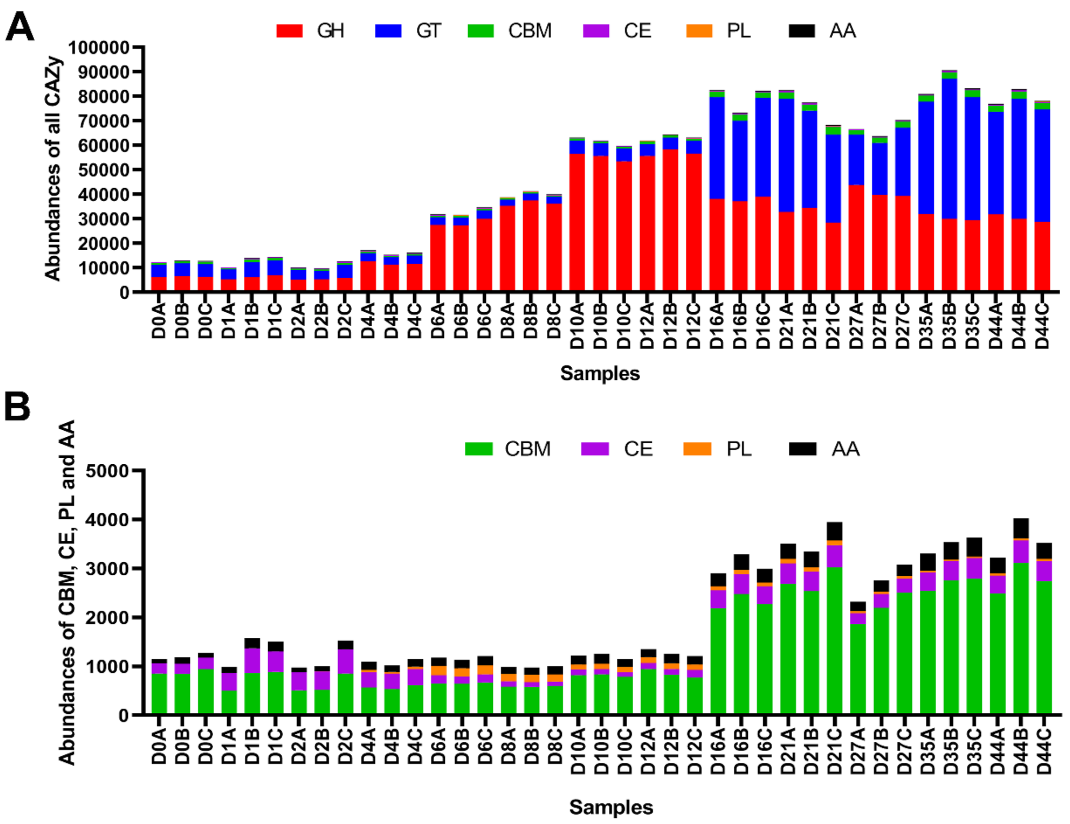

FIG S2 Abundances of the carbohydrate active enzymes (CAZy) in Light-flavor liquor fermentation. (A) Abundances of glycoside hydrolases (GH), glycosyltransferases (GT), carbohydrate-binding modules (CBM), carbohydrate esterases (CE), polysaccharide lyases (PL), auxiliary activities (AA). (B) Abundances of CBM, CE, PL and AA. D0A, D0B and D0C represent the samples isolated from the initial fermentation in pits A, B and C, respectively. D2A, D2B and D2C represent the samples isolated from the 2nd day of fermentation in pits D, E and F, respectively. All samples were named in the same way.

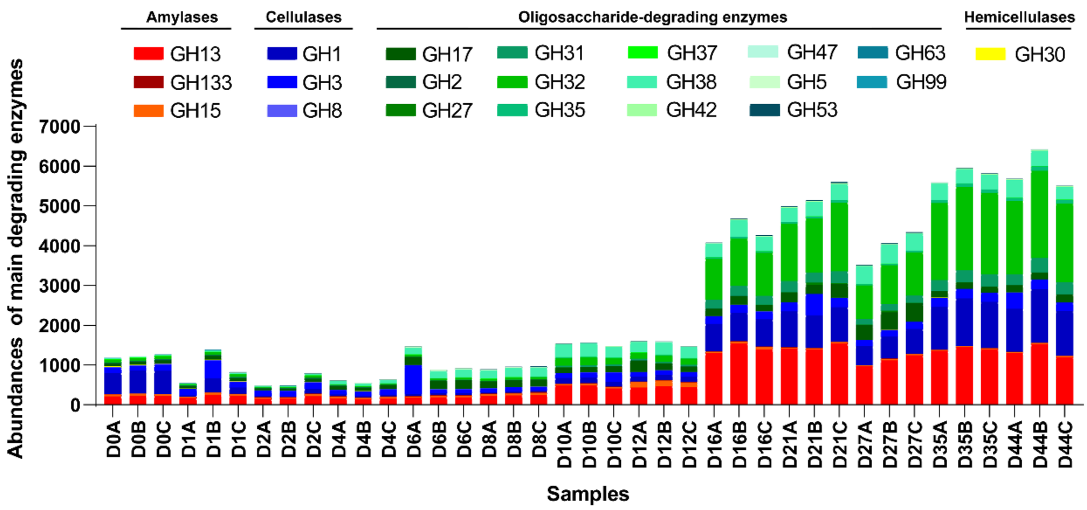

FIG S3 Abundances of the main degrading glycoside hydrolases (GH) family in Light-flavor liquor fermentation. GH were classified by their functions as amylase, cellulase, hemicellulose and oligosaccharide-degrading enzymes. D0A, D0B and D0C represent the samples isolated from the initial fermentation in pits A, B and C, respectively. D2A, D2B and D2C represent the samples isolated from the 2nd day of fermentation in pits D, E and F, respectively. All samples were named in the same way.

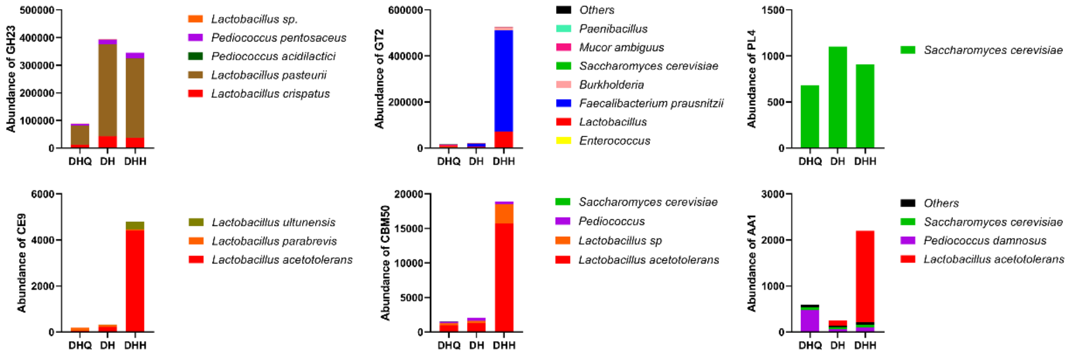

FIG S4 Abundances of the most abundant carbohydrate active enzymes and the contributed microorganisms in Light-flavor liquor fermentation. GH23, glycoside hydrolase family 23; GT2, glycosyltransferase family 2; CBM50, carbohydrate-binding module family 50; CE9, carbohydrate esterase family 9; PL4, polysaccharide lyase family 4; AA1, auxiliary activity family 1. DHQ, DH and DHH represent the stages of early fermentation (0-6 days), middle fermentation (6-12 days) and late fermentation (12-44 days), respectively.

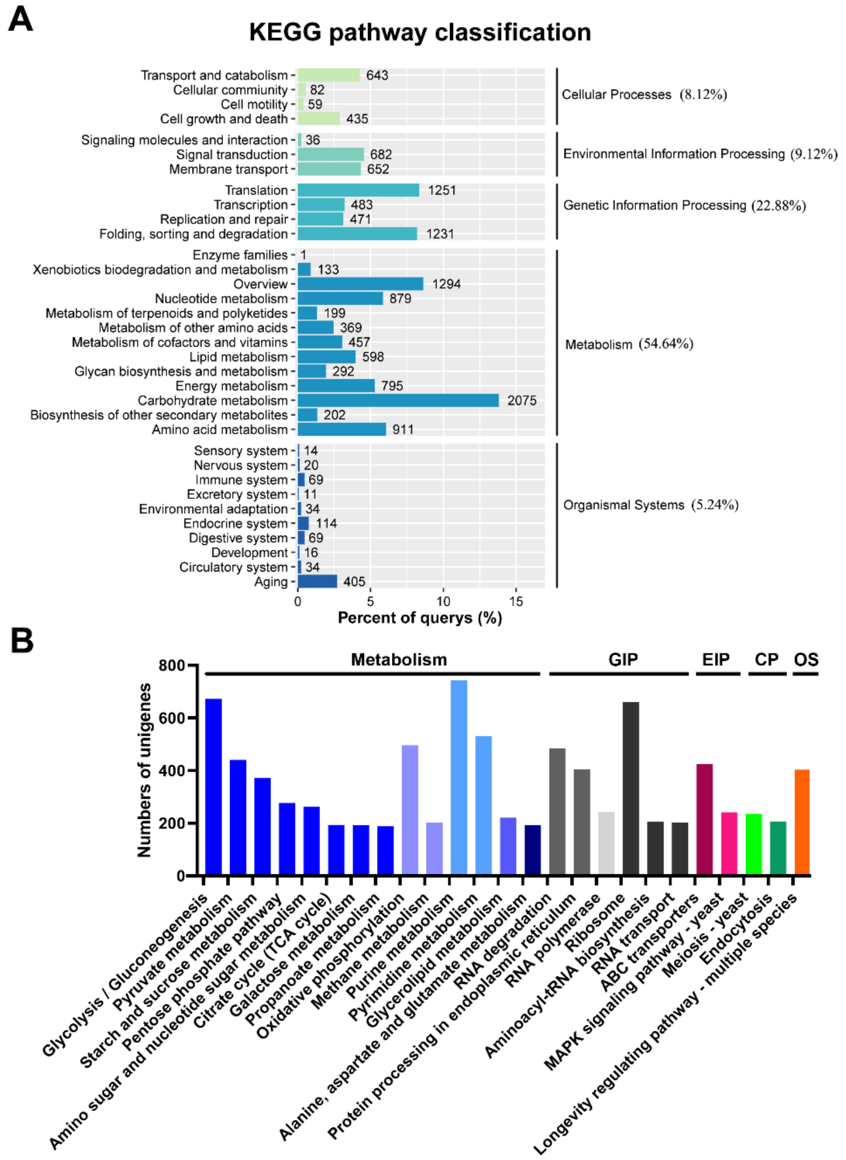

FIG S5 Classification and top 25 KEGG pathways with the largest numbers of unigenes in Light-flavor liquor fermentation. (A) Classification of the KEGG pathways at the levels of organism systems, metabolism, genetic information processing, environmental information processing and cellular processes. (B) Top 25 KEGG pathways with the largest numbers of unigenes. Blue bars represent metabolism; Grey bars represent genetic information processing (GIP); Red bars represent environmental information processing (EIP); Green bars represent cellular processes (CP) and orange bar represents organism systems (OS).

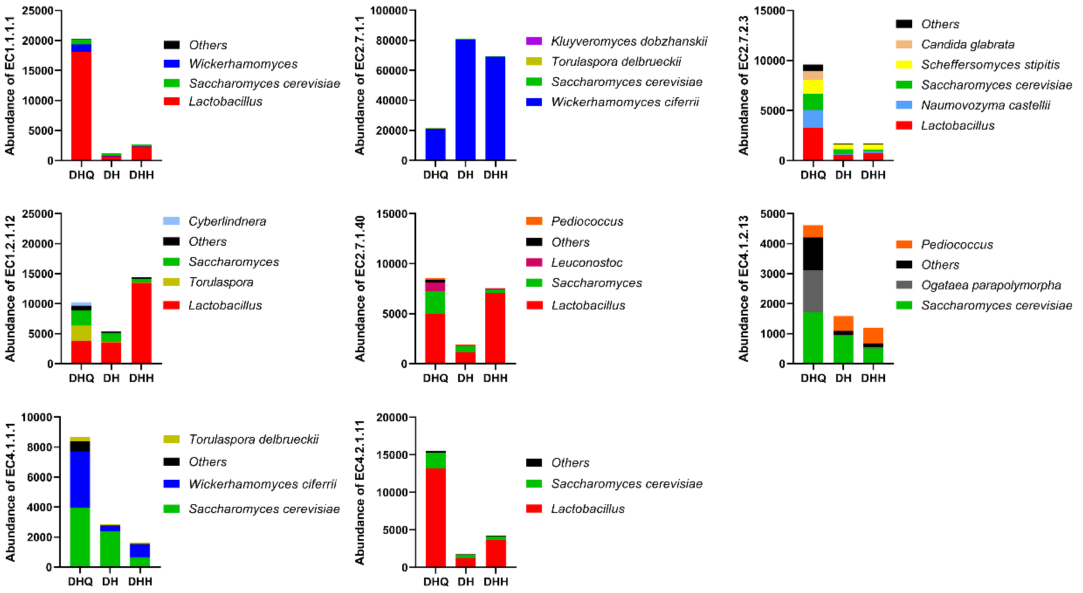

FIG S6 Abundances of the highly expressed enzymes and the contributed microorganisms in the pathway of glycolysis. DHQ, DH and DHH represent the stages of early fermentation (0-6 days), middle fermentation (6-12 days) and late fermentation (12-44 days), respectively. EC1.1.1.1, alcohol dehydrogenase; EC2.7.1.1, hexokinase; EC2.7.2.3, phosphoglycerate kinase; EC4.1.1.1, pyruvate decarboxylase; EC1.2.1.12, glyceraldehyde 3-phosphate dehydrogenase; EC2.7.1.40, pyruvate kinase; EC4.1.2.13, fructose-bisphosphate aldolase; EC4.2.1.11, enolase.

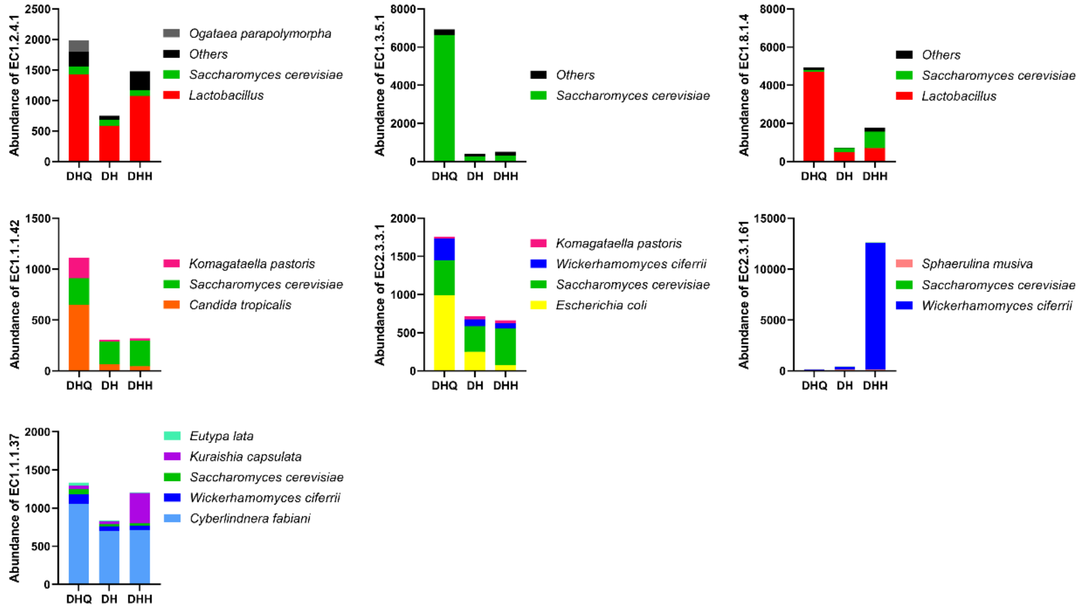

FIG S7 Abundances of the highly expressed enzymes and the contributed microorganisms in the pathway of citrate cycle. DHQ, DH and DHH represent the stages of early fermentation (0-6 days), middle fermentation (6-12 days) and late fermentation (12-44 days), respectively. EC1.2.4.1, pyruvate dehydrogenase; EC2.3.1.61, 2-oxoglutarate dehydrogenase; EC1.3.5.1, succinate dehydrogenase flavoprotein subunit; EC1.8.1.4, dihydrolipoamide dehydrogenase; EC1.1.1.37, malate dehydrogenase; EC1.1.1.42, isocitrate dehydrogenase; EC2.3.3.1, citrate synthase.

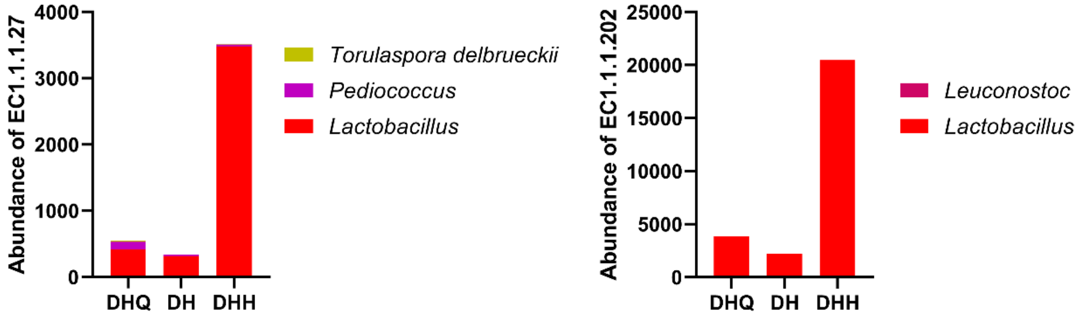

FIG S8 Abundances of the highly expressed enzymes and the contributed microorganisms in the pathway of propanoate metabolism. DHQ, DH and DHH represent the stages of early fermentation (0-6 days), middle fermentation (6-12 days) and late fermentation (12-44 days), respectively. EC1.1.1.27, L-lactate dehydrogenase; EC1.1.1.202, 1, 3-propanediol dehydrogenase.

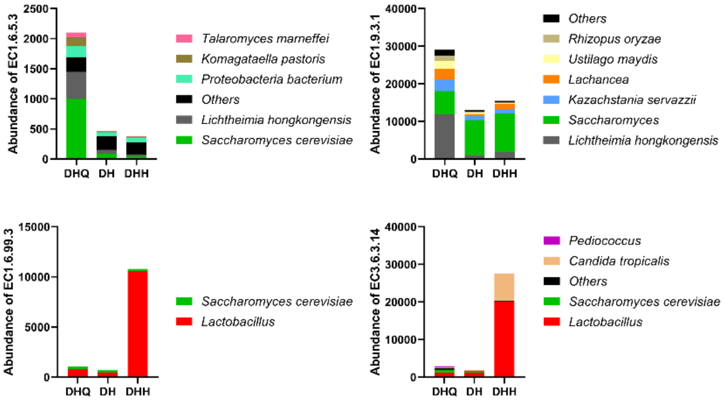

FIG S9 Abundances of the highly expressed enzymes and the contributed microorganisms in the pathway of oxidative phosphorylation. DHQ, DH and DHH represent the stages of early fermentation (0-6 days), middle fermentation (6-12 days) and late fermentation (12-44 days), respectively. EC1.6.5.3 and EC1.6.99.3, NADH dehydrogenase; EC1.9.3.1, cytochrome c oxidase subunit; EC3.6.3.6, H+-transporting ATPase; EC3.6.3.14, F-type H+-transporting ATPase subunit.

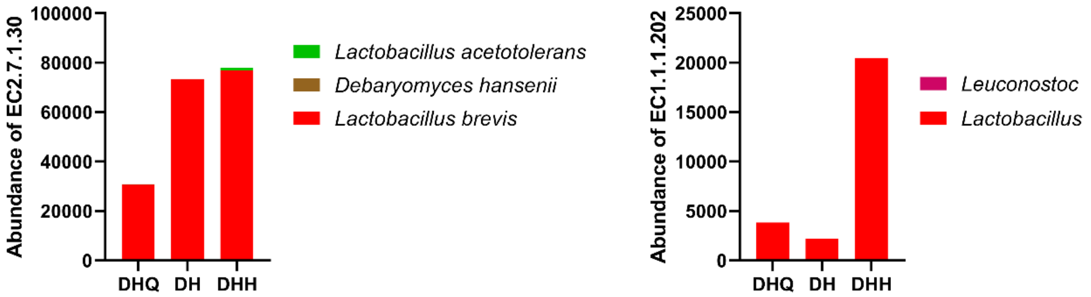

FIG S10 Abundances of the highly expressed enzymes and the contributed microorganisms in the pathway of glycerolipid metabolism. DHQ, DH and DHH represent the stages of early fermentation (0-6 days), middle fermentation (6-12 days) and late fermentation (12-44 days), respectively. EC2.7.1.30, glycerol kinase; EC1.1.1.202, 1, 3-propanediol dehydrogenase.

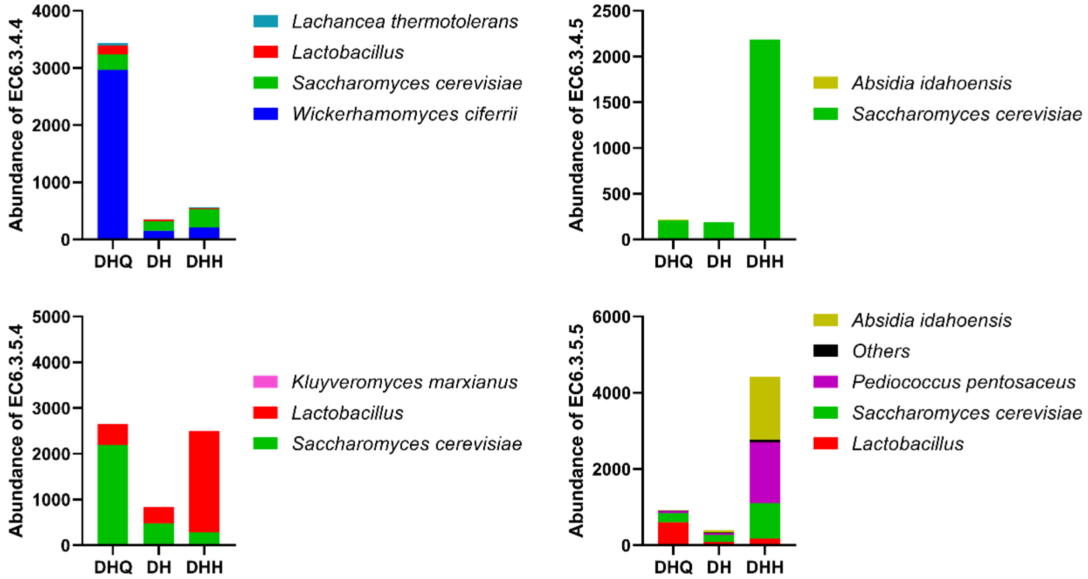

FIG S11 Abundances of the highly expressed enzymes and the contributed microorganisms in the pathway of alanine, aspartate and glutamate metabolism. DHQ, DH and DHH represent the stages of early fermentation (0-6 days), middle fermentation (6-12 days) and late fermentation (12-44 days), respectively. EC6.3.4.4, adenylosuccinate synthase; EC6.3.4.5, argininosuccinate synthase; EC6.3.5.4, asparagine synthase; EC6.3.5.5, carbamoyl-phosphate synthase.

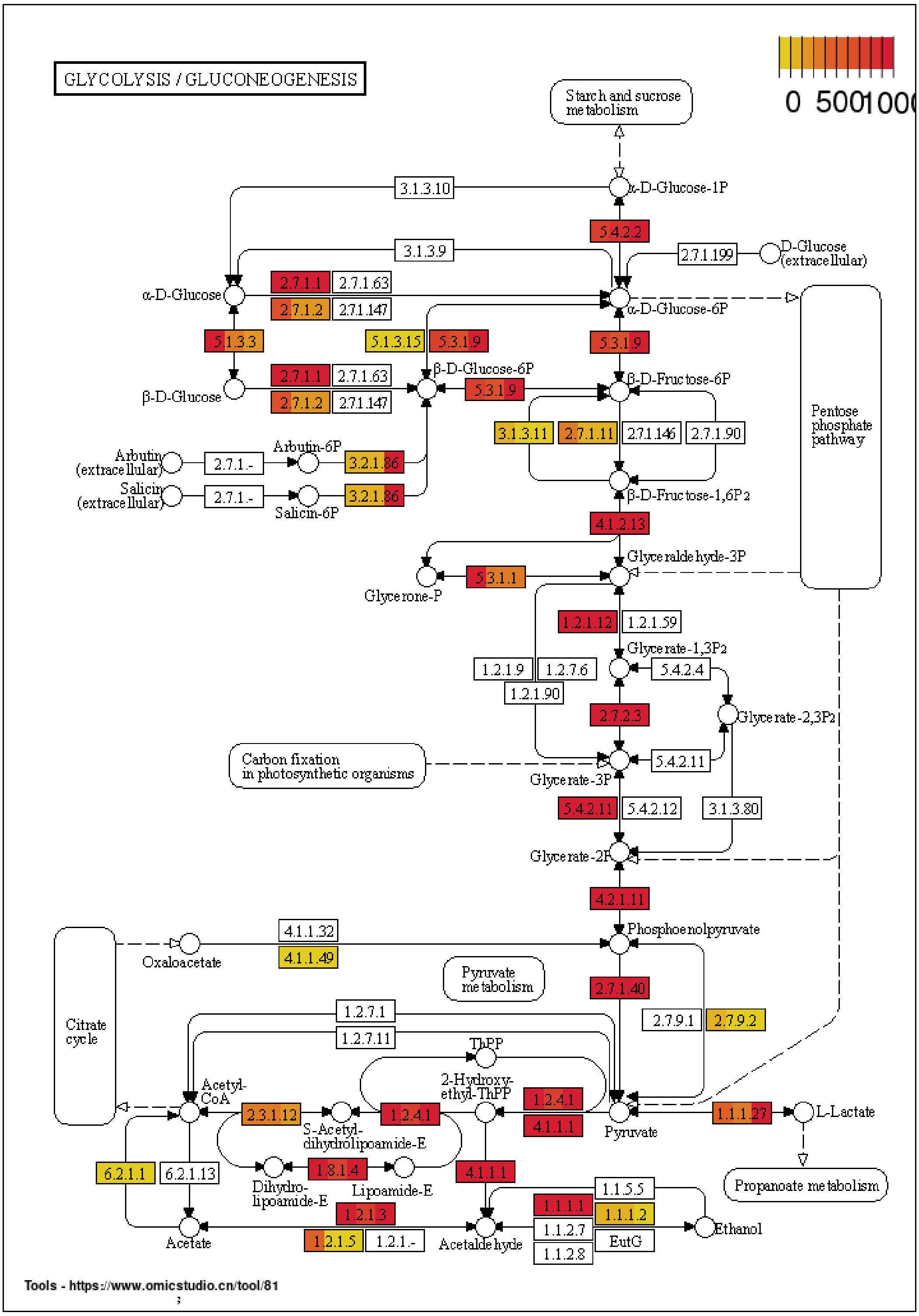

FIG S12 Highly expressed enzymes marked in the pathway of glycolysis at three fermentation stages. From left to right, three parts of color bars represent the stages of early fermentation (0-6 days), middle fermentation (6-12 days) and late fermentation (12-44 days), respectively. The color indicates the enzyme expression level based on the abundances of unigenes.

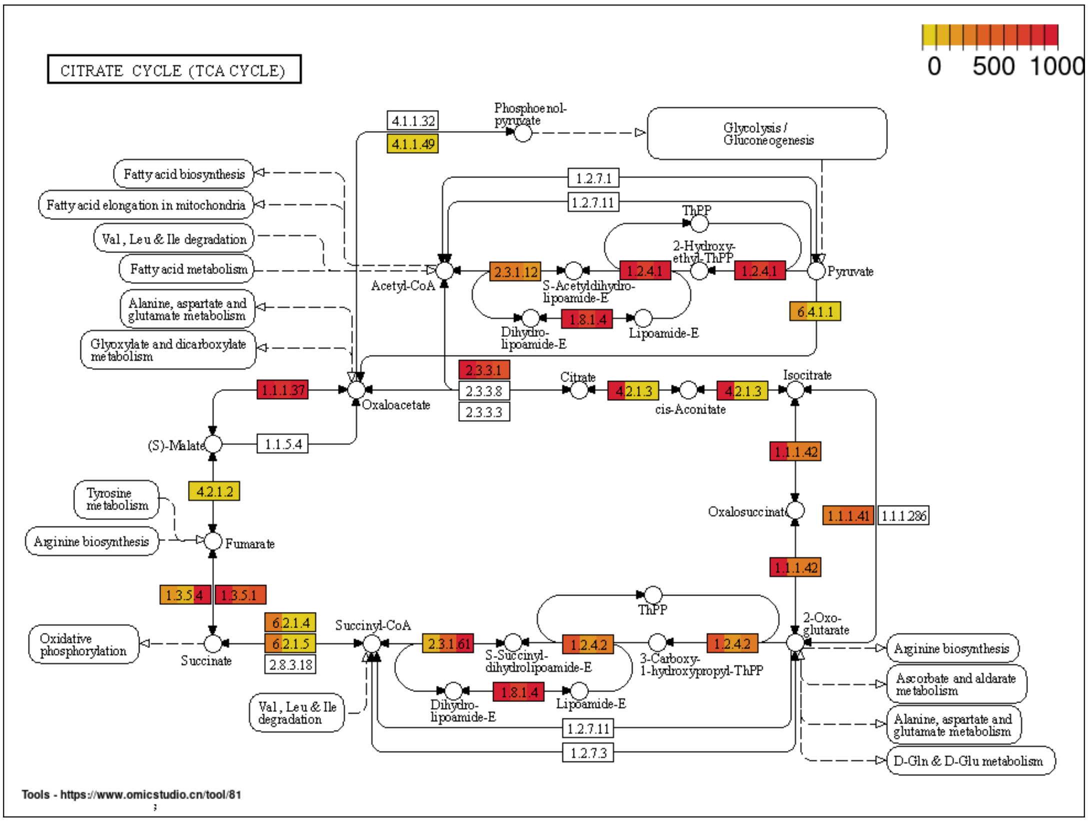

FIG S13 Highly expressed enzymes marked in the pathway of TCA cycle at three fermentation stages. From left to right, three parts of color bars represent the stages of early fermentation (0-6 days), middle fermentation (6-12 days) and late fermentation (12-44 days), respectively. The color indicates the enzyme expression level based on the abundances of unigenes.

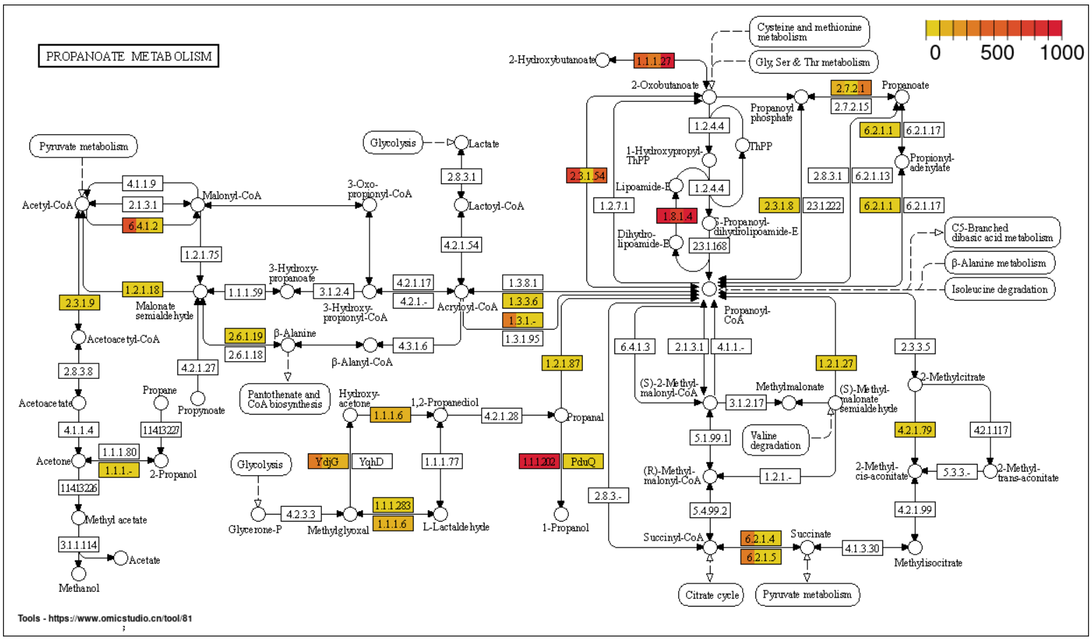

FIG S14 Highly expressed enzymes marked in the pathway of propanoate metabolism at three fermentation stages. From left to right, three parts of color bars represent the stages of early fermentation (0-6 days), middle fermentation (6-12 days) and late fermentation (12-44 days), respectively. The color indicates the enzyme expression level based on the abundances of unigenes.

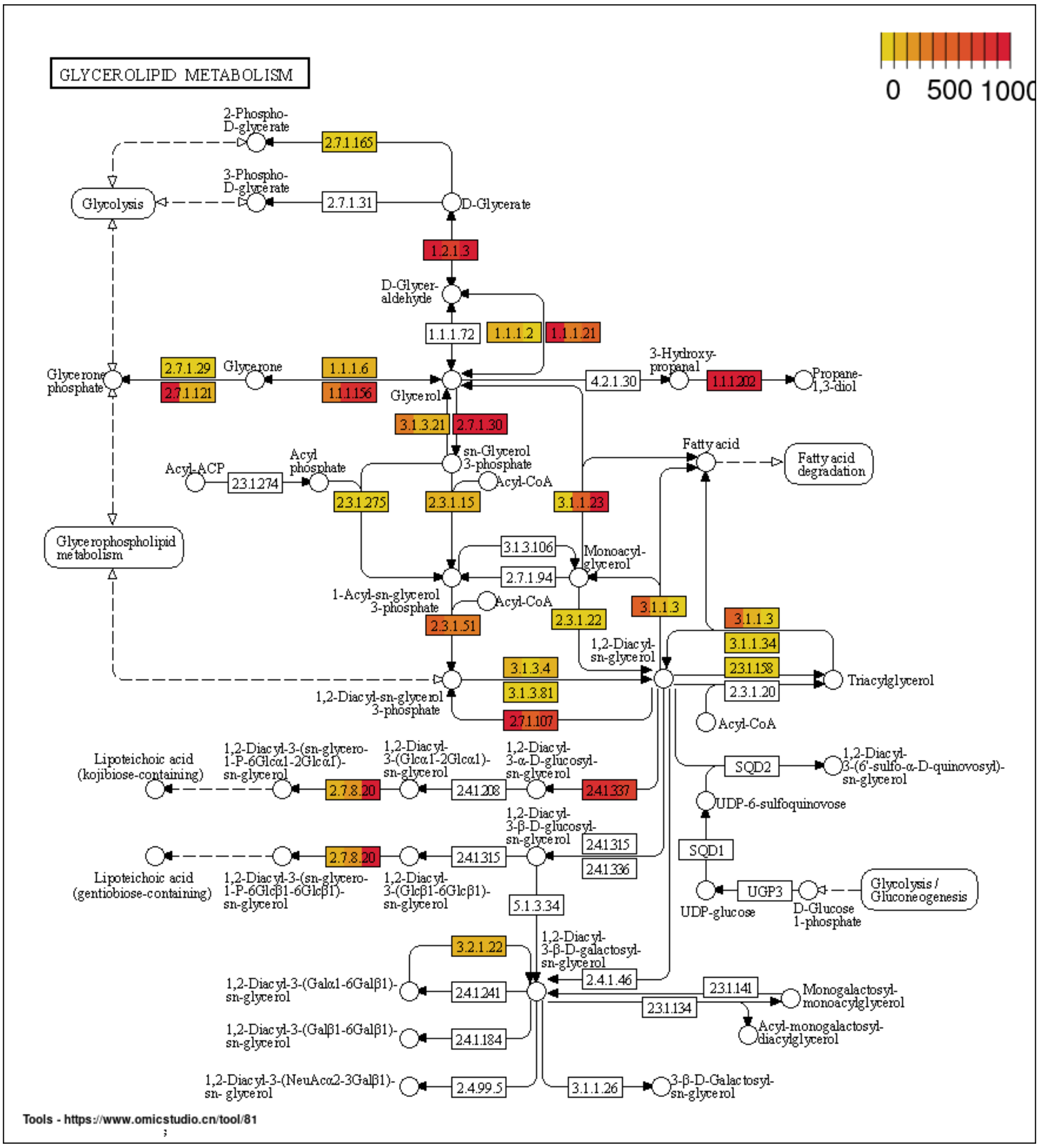

FIG S15 Highly expressed enzymes marked in the pathway of glycerolipid metabolism at three fermentation stages. From left to right, three parts of color bars represent the stages of early fermentation (0-6 days), middle fermentation (6-12 days) and late fermentation (12-44 days), respectively. The color indicates the enzyme expression level based on the abundances of unigenes.

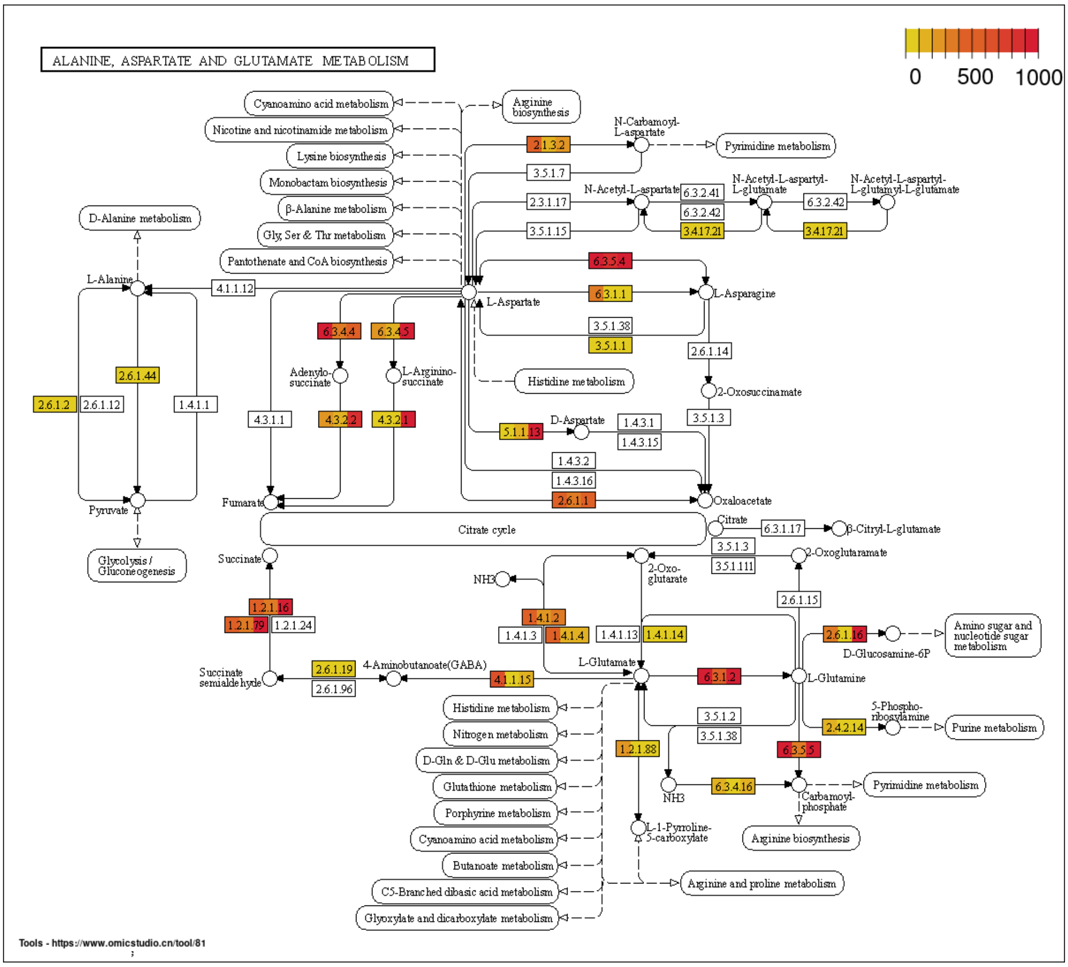

FIG S16 Highly expressed enzymes marked in the pathway of alanine, aspartate and glutamate metabolism at three fermentation stages. From left to right, three parts of color bars represent the stages of early fermentation (0-6 days), middle fermentation (6-12 days) and late fermentation (12-44 days), respectively. The color indicates the enzyme expression level based on the abundances of unigenes.

Time	A	B	C	D	E	F	G
D0	√	√	√				FS
D1	√	√	√				FS
D2				√	√	√	FS
D4	√	√	√				FS
D6				√	√	√	FS
D8	√	√	√				FS
D10				√	√	√	FS
D12	√	√	√				FS
D16				√	√	√	FS
D21	√	√	√				FS
D27				√	√	√	FS
D35	√	√	√				FS
D44				√	√	√	FS

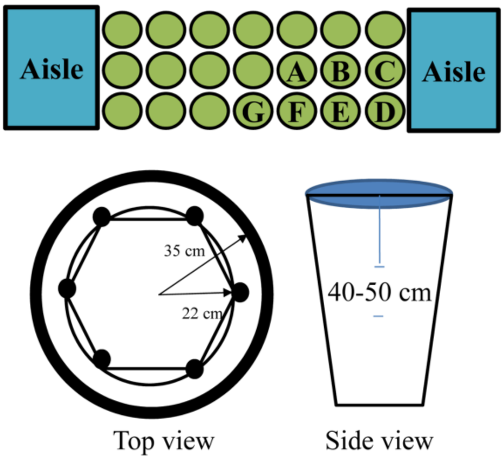

FIG S17 Time points and methods of the samples collected from the fermentation pits. D0 represents the samples isolated from the fermentation start time. Samples from A, B and C pits were named as D0A, D0B and D0C, respectively. D2 represents the samples isolated from the 2nd day of fermentation. Samples from D, E and F pits were named as D2A, D2B and D2C, respectively. The following samples were named in the same way. Samples located between 40 and 50 cm in the pits were collected and mixed well immediately for use, and then supply the same amount of feedstock from pit G to the sampling spaces. FS, Feedstock supply pit; cm, centimeter.
